# Enzyme cascades for nucleotide sugar regeneration in glycoconjugate synthesis

**DOI:** 10.1007/s00253-025-13432-2

**Published:** 2025-02-27

**Authors:** Lothar Elling

**Affiliations:** Laboratory for Biomaterials, Institute of Biotechnology, and Helmholtz-Institute for Biomedical Engineering, RWTH Aachen University, Pauwelsstraße 20, 52074 Aachen, Germany

**Keywords:** Nucleotide sugars, In situ regeneration, Leloir glycosyltransferases, Sucrose synthase, Glycoconjugates, Natural products

## Abstract

**Abstract:**

Leloir glycosyltransferases are instrumental in the synthesis of glycoconjugates. Nucleotide sugars as their donor substrates are still considered expensive making preparative enzymatic syntheses economically unattractive. The review highlights the development and advancements of in situ regeneration cycles that utilize nucleotides as byproducts from glycosyltransferase reactions to synthesize respective nucleotide sugars. This approach reduces costs and avoids inhibition of Leloir glycosyltransferases. Regeneration cycles for ten nucleotide sugars are explored emphasizing enzyme cascades from salvage pathways and nucleotide biosynthesis. Additionally, the review highlights advancements involving sucrose synthase for the in situ regeneration of nucleotide sugars from sucrose. Sucrose synthase as the first example of a reversible glycosyltransferase reaction paved the way to establish economic syntheses of glycosylated natural products. Important aspects like enzyme immobilization and protein fusion to optimize processes are discussed. Overall, the review underscores the significance of advanced in situ regeneration cycles for nucleotide sugars for cost-effective access to high-value glycoconjugates.

**Key points:**

•* Enzyme cascades for in situ regeneration of nucleotide sugars*

•* Effective cycles for large-scale synthesis of glycoconjugates*

•* Regeneration of nucleotide sugars from sucrose by sucrose synthase*

**Graphical abstract:**

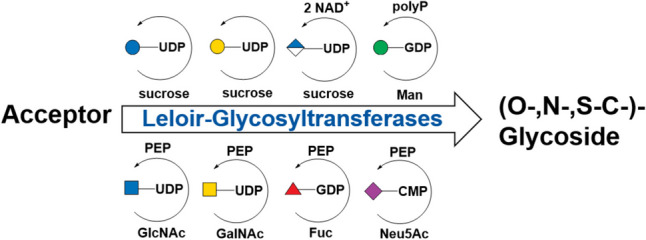

**Supplementary Information:**

The online version contains supplementary material available at 10.1007/s00253-025-13432-2.

## Introduction

### History

**Fig. 1:**
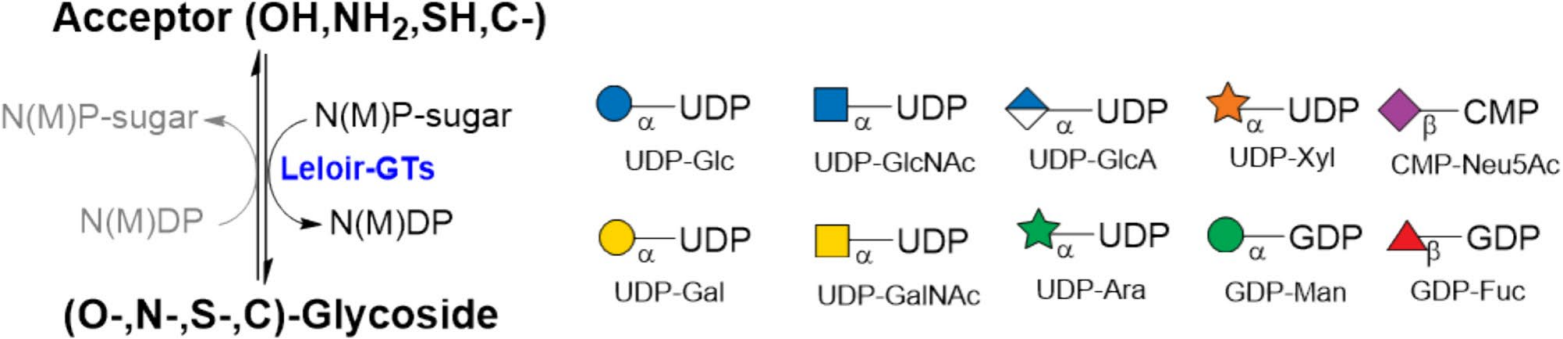
General scheme for Leloir GT reactions. An acceptor is glycosylated by the transfer of the sugar from the respective nucleotide sugar yielding the glycoside and the respective nucleotide as products. Some Leloir GTs show reversible reactions (in grey) rendering nucleotide sugars from glycosides and nucleotides. Common nucleotide sugars are depicted according to the symbol nomenclature for graphical representations of glycans (Varki et al. 2015) (see Scheme S1 in SI for chemical structures, names, and symbols)

Luis F. Leloir was awarded the Nobel Prize in Chemistry in 1970 for the discovery of nucleotide sugars and their role in the biosynthesis of carbohydrates (Figueroa et al. 2021). He and his colleagues identified the nucleotide sugars UDP-Glc and UDP-Gal as essential metabolites in plant metabolism. They elucidated the role of glycosyltransferases in biosynthetic pathways for sucrose and starch and established a foundation for understanding the biosynthesis of plant polysaccharides and signaling molecules such as trehalose-6-phosphate. Accordingly, glycosyltransferases using nucleotide sugars as donor substrates are termed Leloir glycosyltransferases (Fig. 1) (CAZyPedia: https://www.cazypedia.org/index.php/Glycosyltransferases).

#### Enzymatic synthesis of glycoconjugates with Leloir glycosyltransferases

In the field of chemistry, the synthesis of glycans involves a series of synthetic steps conducted in organic solvents, which often result in yields that are relatively low to moderate (Malik et al. 2021). The concept of utilizing Leloir GTs and nucleotide sugars was first proposed in 1980, with the synthesis of *N*-acetyllactosamine (Gal(β1–4)GlcNAc, LacNAc; LN2) (Nunez and Barker 1980). LacNAc serves as the fundamental building block for glycan epitopes, including those associated with the AB0 and Lewis blood groups. However, the requisite donor substrate, UDP-Gal, had to be synthesized via chemical means. Furthermore, β1,4-galactosyltransferase (β4GalT), derived from bovine or human milk, was purified through UDP-, UMP-, or GlcNAc-agarose affinity chromatography (Barker et al. 1972).

**Fig. 2:**
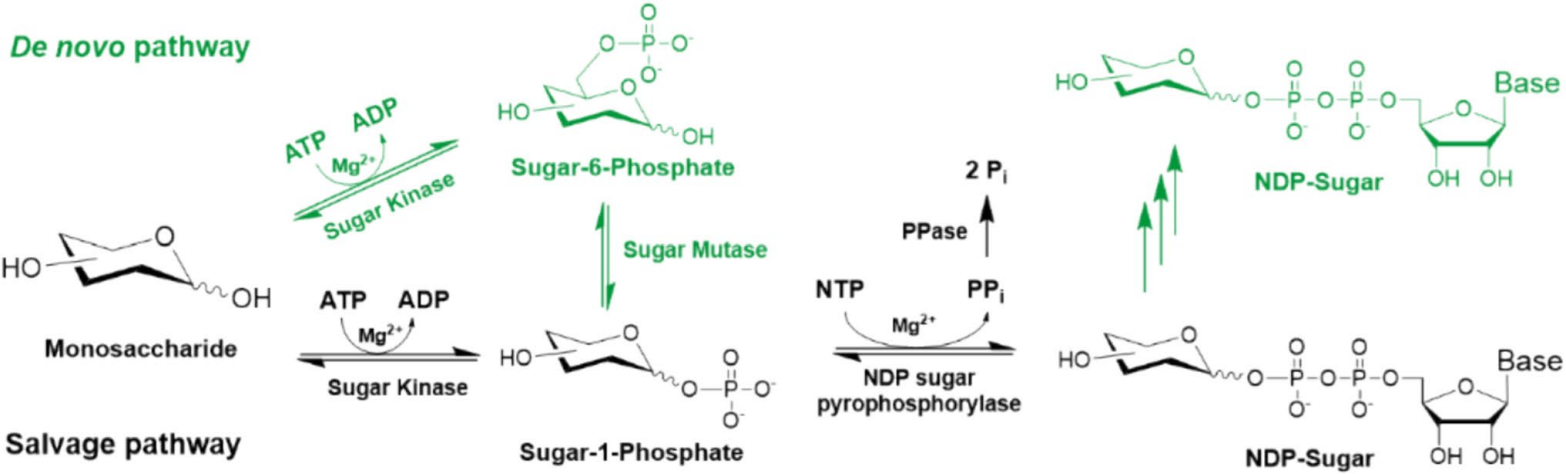
De novo (green/black) and salvage pathway (black) for nucleotide biosynthesis. The de novo includes enzymes for the synthesis of sugar 6-phosphates (sugar kinase), the conversion to sugar-1-phosphates (sugar mutase), and further modifications of NDP-sugars (green arrows). The salvage pathway is depicted in black and includes sugar-1-P kinases and NDP-sugar pyrophosphorylases

For the larger-scale enzymatic synthesis of glycoconjugates, two key issues require solutions: the accessibility of nucleotide sugars and the availability of Leloir glycosyltransferases; the chemical synthesis of nucleotide sugars was successfully achieved on a gram-scale basis (Baisch and Öhrlein 1997). Nevertheless, the first multi-gram-scale enzymatic synthesis of nucleotide sugars was achieved through biotransformation with permeabilized microbial cells, although the yields for product purification remained low (Tabata et al. 2000; Koizumi et al. 2000; Endo et al. 2000; Endo and Koizumi 2000; Endo et al. 1999; Koizumi et al. 1998). The accessibility of a vast array of Leloir glycosyltransferases was constrained by the intrinsic difficulties encountered in the generation of eukaryotic Leloir GTs in recombinant microbial hosts. Nevertheless, all these issues have been successfully addressed. Nucleotide sugars are produced in multi-gram quantities by robust enzyme cascades created by nucleotide sugar pathway enzymes (Fig. 2) (Frohnmeyer and Elling 2023; Frohnmeyer et al. 2024; Zheng et al. 2022; Li et al. 2021). Pro- and eukaryotic Leloir GTs are produced in appropriate hosts, including *E. coli*, yeast, insect, and mammalian cell lines (Moremen et al. 2017; Nidetzky et al. 2018; Jaroentomeechai et al. 2022; Hussnaetter et al. 2023).

Instead of producing nucleotide sugars in a separate process, the in situ generation of nucleotide sugars by the combination of enzyme cascades with Leloir GTs has enabled the one-pot synthesis of glycoconjugates (HMOS, glycosphingolipids, glycopeptides) in quantities up to multi-gram amounts (Hussnaetter et al. 2023; Chen 2024; Bai et al. 2022).

A more economical way to provide nucleotide sugar is based on in situ regeneration cycles utilizing the nucleotide by-product from a glycosyltransferase (GT) reaction for the synthesis of the respective nucleotide sugar. In this way, the inhibition of GTs by the released nucleoside di-/monophosphate (NDP/NMP) is avoided and costs for nucleotide sugars are reduced. The regeneration cycles include enzyme cascades for the synthesis of nucleoside triphosphates (NTPs) from NDP or NMP, enzymes for nucleotide sugar synthesis, and, in some cases, enzymes for further conversion of the nucleotide sugar.

**Fig. 3:**
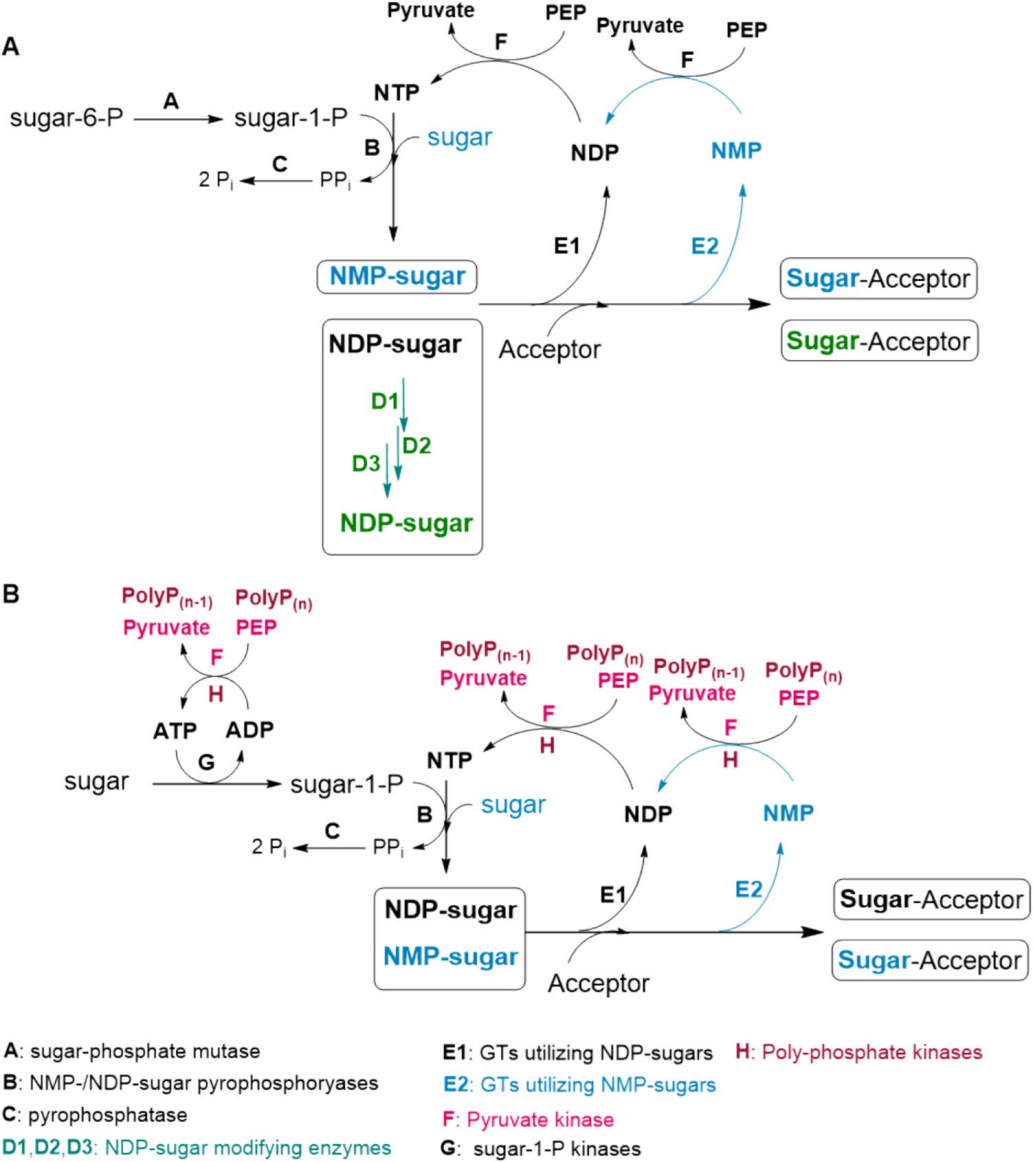
General schemes for in situ nucleotide sugar regeneration NDP-sugars (green/black) and NMP-sugars (blue). **A** De novo pathway enzymes (green) and pyruvate kinase (PK)/phosphoenolpyruvate (PEP) system for nucleotide phosphorylation; **B** salvage pathway enzymes and PK/PEP or poly-phosphate (polyP)/polyP kinases (PPK) system for nucleotide phosphorylation

This review summarizes advances and applications of in situ nucleotide sugar regeneration cycles for the synthesis of glycoconjugates. Figure 3 depicts the evolvement of in situ nucleotide sugar regeneration cycles including enzymes from de novo and salvage pathways and enzymes for nucleotide regeneration. Table S1 (Supplementary Information) summarizes all nucleotide sugar regeneration cycles.

### Enzyme cascades for NDP/NMP-sugar regeneration with de novo and salvage pathway enzymes

#### UDP-Glc/UDP-Gal

**Fig. 4:**
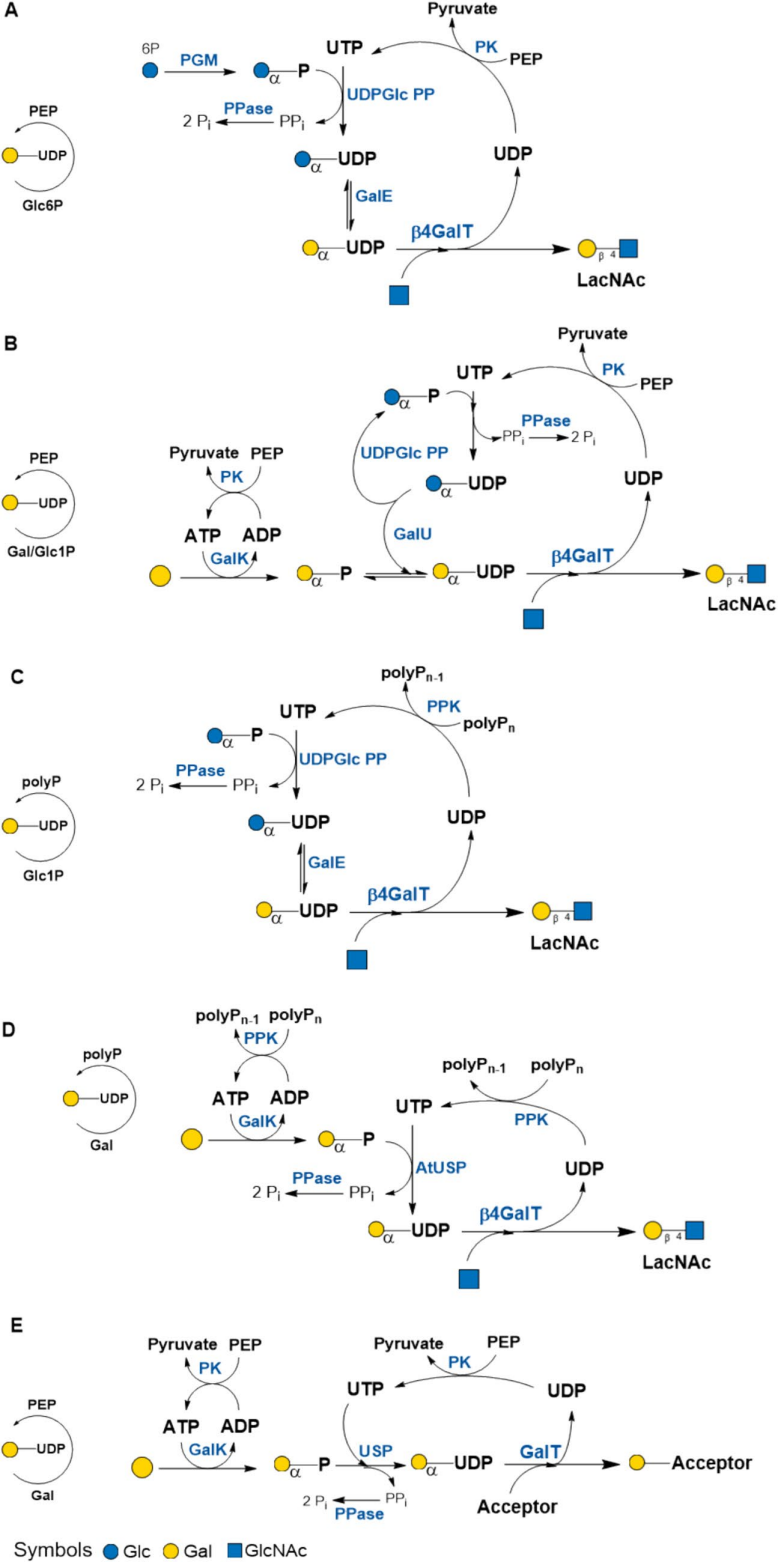
Synthesis of LacNAc with UDP-Gal regeneration cycles. **A** UDP-GlcPP, GalE, and PK/PEP; **B** UDP-Glc PP, GalU, and PK/PEP; **C** UDP-Glc PP, GalE, and PPK/polyP_n_. **D** Salvage pathway enzymes: GalK, *At*USP, and PPK/polyP_n_. **E** In situ regeneration of UDP-Gal with salvage pathway enzymes and PK/PEP. The regeneration cycles for the individual nucleotide sugars are symbolized by circles including the starting substrate, the substrate for nucleotide regeneration, and further cofactors (see also Table S1; SI). PGM: phosphoglucomutase; UDP-Glc PP: UDP-Glc pyrophosphorylase; GalE: UDP-Glc 4’-epimerase; GalU: UDP-Gal uridylyltransferase GalK: galactokinase; β4GalT: β1,4-galactosyltransferase; PK: pyruvate kinase; PPK: polyP_n_ kinase, USP: UDP-sugar pyrophosphorylase

The first regeneration cycle of a nucleotide sugar was published by Wong et al. (1982). In their pioneering work, they synthesized the disaccharide *N*-acetyllactosamine (LacNAc) type 2 with β4GalT and in situ regeneration of UDP-Gal (Fig. 4A). The synthetic cycle started from Glc-6-phosphate and *N*-acetylglucosamine (GlcNAc) including six enzymes: β4GalT; pyruvate kinase (PK), phosphoglucomutase (PGM), UDP-Glc pyrophosphorylase (UDP-Glc PP), pyrophosphatase (PPase), and UDP-Glc 4’-epimerase (GalE). The LacNAc synthesis was performed on a multi-g scale with immobilized enzymes: 34 mmol (13 g) LacNAc was obtained with 85% yield after synthesis and 70% yield after product isolation. 0.5 mmol UDP and 42 mmol PEP were applied for nucleotide regeneration with PK; the cycle number for UDP-Gal was 68. The authors concluded their study as follows: “The principles underlying these procedures should be applicable to the several different nucleoside diphosphate sugars required in other polysaccharide synthesis” (Wong et al. 1982). The UDP-Gal regeneration cycle (or “cyclic multi-enzyme system for galactosylation”) with immobilized enzymes was further exploited for the synthesis of more complex glycan structures like the I-blood group antigen (tetrasaccharide with branched LacNAc units) (Augé et al. 1986). Omitting GalE rendered in situ regeneration of UDP-Glc with a cycle number of 10 for the synthesis of sucrose and trehalose by immobilized sucrose synthase and trehalose synthase, respectively (Haynie and Whitesides 1990).

The synthesis of UDP-Glc is a pivotal step in the regeneration of UDP-Gal via enzymes derived from the Leloir biosynthetic pathway. UDP-Glc 4'-epimerase (GalE) (Fig. 4A) and Gal-1-P uridylyltransferase (GalU) (Fig. 4B) are both enzymes that utilize UDP-Glc as a substrate (Wong et al. 1992). GalU uses Gal-1-P, which is produced from Gal by galactokinase (GalK). However, the phosphorylation of UDP using PEP represents a substantial cost factor. The substitution of the pyruvate kinase (PK)/PEP system with polyphosphate kinase (PPK)/polyphosphate (polyP_n_) provides a more cost-effective approach (Fig. 4C and D) (Noguchi and Shiba 1998; Jiao et al. 2024). It is noteworthy that the process did not include pyrophosphatase (PPase), which resulted in a low LacNAc yield of approximately 65%. Nevertheless, the study demonstrates that β4GalT is active in the presence of 75 mM polyphosphate (Noguchi and Shiba 1998). Including enzymes from the salvage pathway for UDP-Gal starts the synthesis of LacNAc from the monosaccharides Gal and GlcNAc with cost effective nucleotide synthesis using PPK/polyP (Fig. 4D) (Jiao et al. 2024). Salvage pathway enzymes for UDP-Gal synthesis (Fig. 4E) together with further nucleotide sugar regeneration cycles were employed for the synthesis of tumor-associated antigens (Figure S4) (Tsai et al. 2013; Wu et al. 2019).

#### UDP-GlcA

**Fig. 5:**
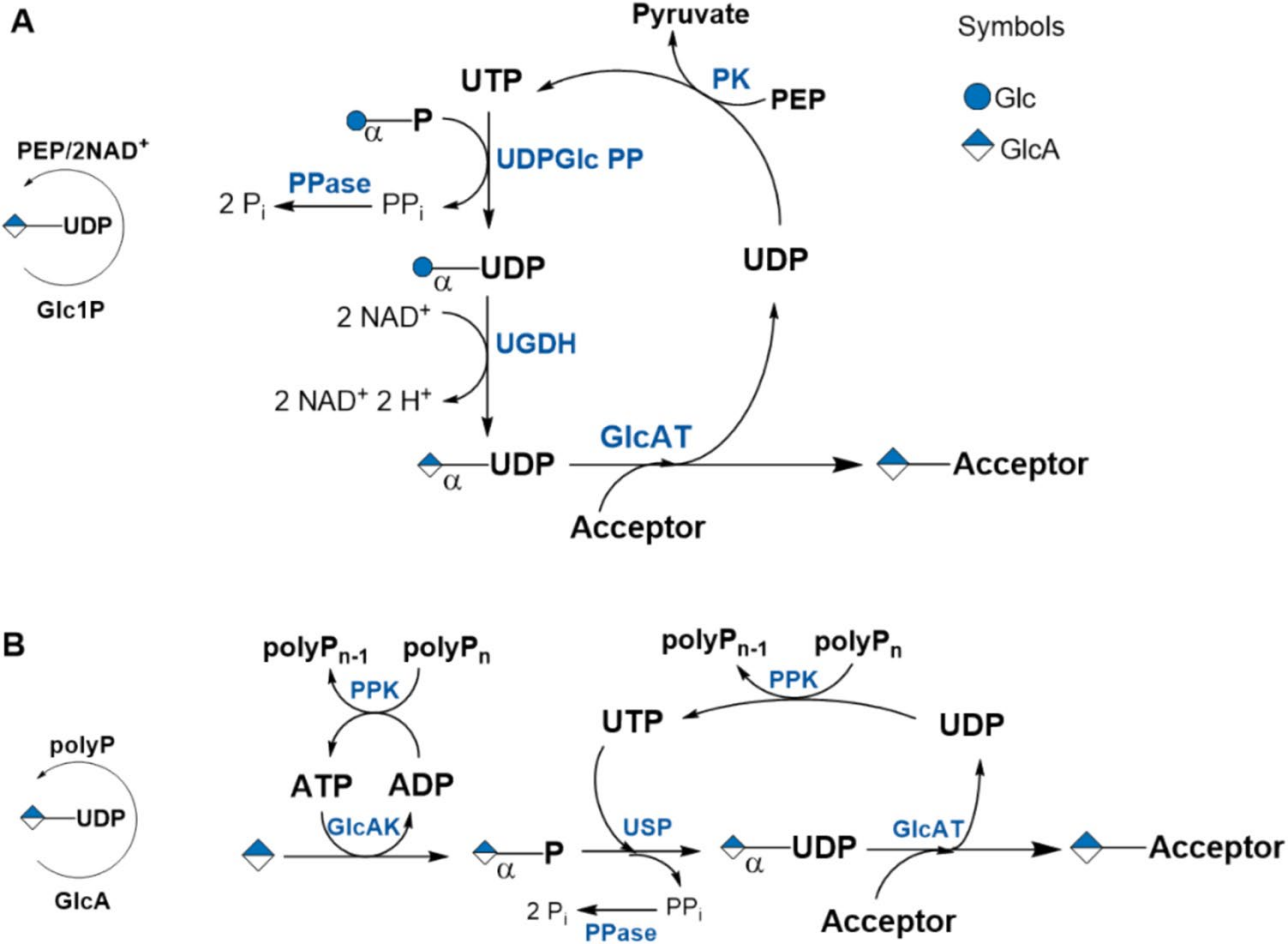
**A** In situ regeneration cycle for UDP-GlcA from UDP-Glc for the synthesis of β-d-glucuronides by glucuronosyltransferases (GlcATs) (Gygax et al. 1991); **B** general scheme for UDP-GlcA regeneration with salvage pathway enzymes (Gottschalk et al. 2019) (see also Table S1; SI). PGM: phosphoglucomutase; UDP-Glc PP: UDP-Glc pyrophosphorylase; UGDH: UDP-Glc dehydrogenase; GlcAT: glucuronosyltransferase; PK: pyruvate kinase; GlcAK: glucuronic acid-1-phosphate kinase; USP: UDP-sugar pyrophosphorylase. PPK: polyP kinase

**Fig. 6:**
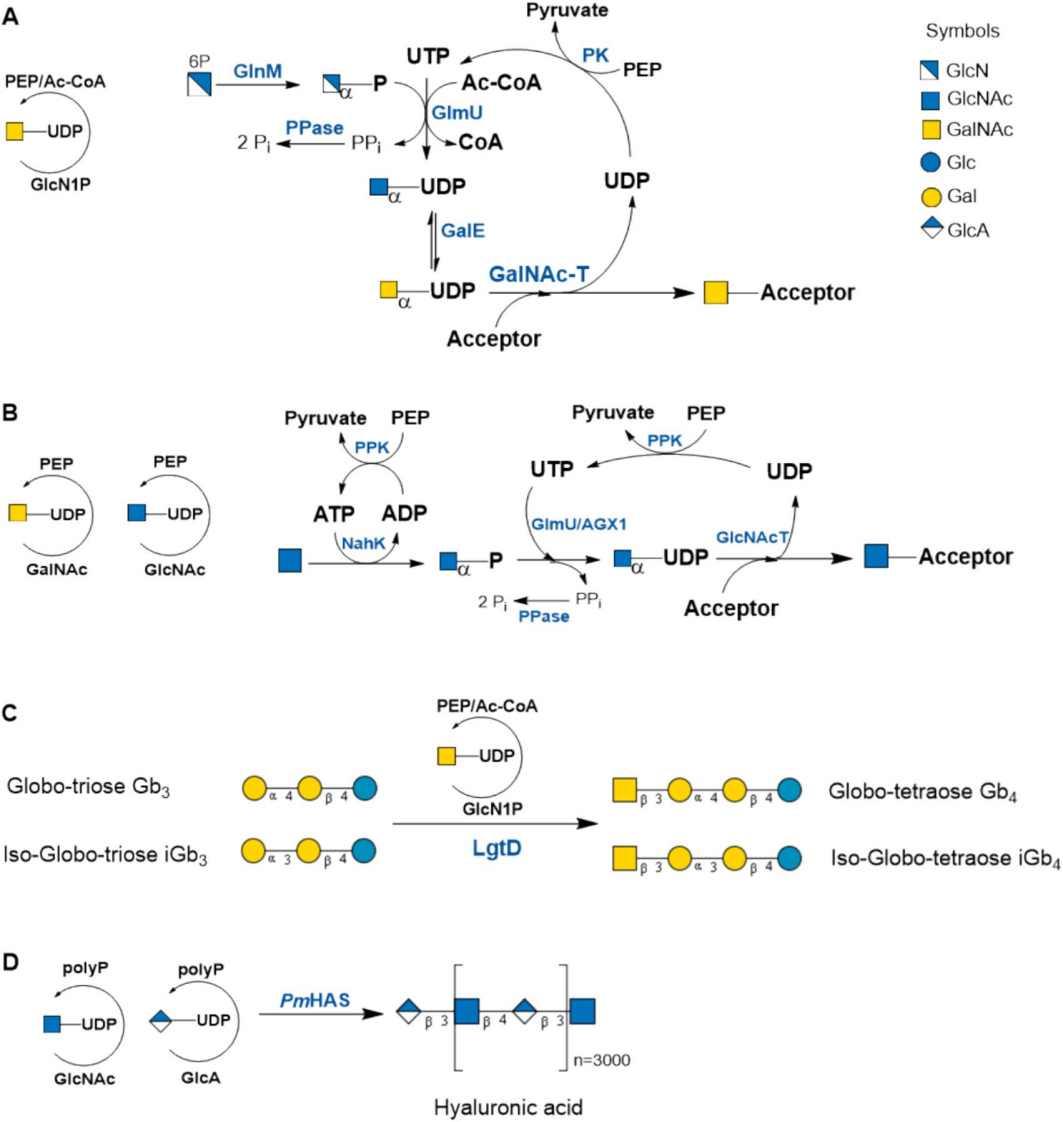
**A** Regeneration cycle for UDP-GalNAc. The cofactor Ac-CoA is regenerated with phosphate acetyltransferase using acetyl-phosphate (not shown). **B** Salvage pathway for the regeneration of UDP-GlcNAc/GalNAc. The enzymes NahK and GlmU/AGX1 are promiscuous to convert GlcNAc and GalNAc. **C** Synthesis of the glycosphingolipid glycans Gb_4_ and Iso-Gb_4_ and **D** hyaluronic acid is depicted (see also Table S1; SI)

The in situ regeneration of uridine 5’-diphosphoglucuronic acid (UDP-GlcA) was reported using a multi-enzyme system and a liver homogenate. UDP-GlcA was generated by UDP-Glc dehydrogenase (UGDH) for the oxidation of UDP-Glc (Fig. 5A). The study highlights the broad aglycon acceptance of liver UDP-glucuronosyltransferases (GlcATs) and the one-pot reaction offers a cost-effective and practical approach for the enzymatic synthesis of β-d-glucuronides (Gygax et al. 1991). UDP-GlcA regeneration with salvage pathway enzymes including GlcA kinase (GlcAK) and USP, both from *Arabidopsis thaliana*, was established and used in the enzymatic synthesis of hyaluronan (Figs. 5B and 6D) (Gottschalk et al. 2019).

#### UDP-GlcNAc/UDP-GalNAc

Regeneration cycles for UDP-*N*-acetyl-glucosamine (UDP-GlcNAc) and UDP-*N*-acetyl-galactosamine (UDP-GalNAc) start from either glucosamine-6-P (Shao et al. 2002) (Fig. 6A) or GlcNAc-1-P (Figure S1A). Including a UDP-GlcNAc 4’-epimerase (GalE) in the enzyme cascade renders UDP-GalNAc from UDP-GlcNAc. The glycosphingolipid glycans Globotetraose (Gb_4_), GalNAc(β1–3)Gal(α1–4)Gal(β1–4)Glc, and iso-globotetraose (iGb_4_), GalNAc(β1–3)Gal(α1–3)Gal(β1–4)Glc, were synthesized with in situ regeneration of UDP-GalNAc (Shao et al. 2002) (Fig. 6C). The combination of the bacterial HexNAc-1-phosphate kinase (NahK) with either the bacterial UDP-GlcNAc/GalNAc pyrophosphorylase from bacteria (GlmU) or human (AGX1) opened the in situ regeneration of UDP-GlcNAc/UDP-GalNAc by a salvage pathway (Frohnmeyer and Elling 2023) (Fig. 6B). UDP-GlcNAc regeneration was utilized in the synthesis of hyaluronan (Gottschalk et al. 2019; De Luca et al. 1995) (Fig. 6D and Figure S1B).

#### GDP-Man/GDP-Fuc

**Fig. 7:**
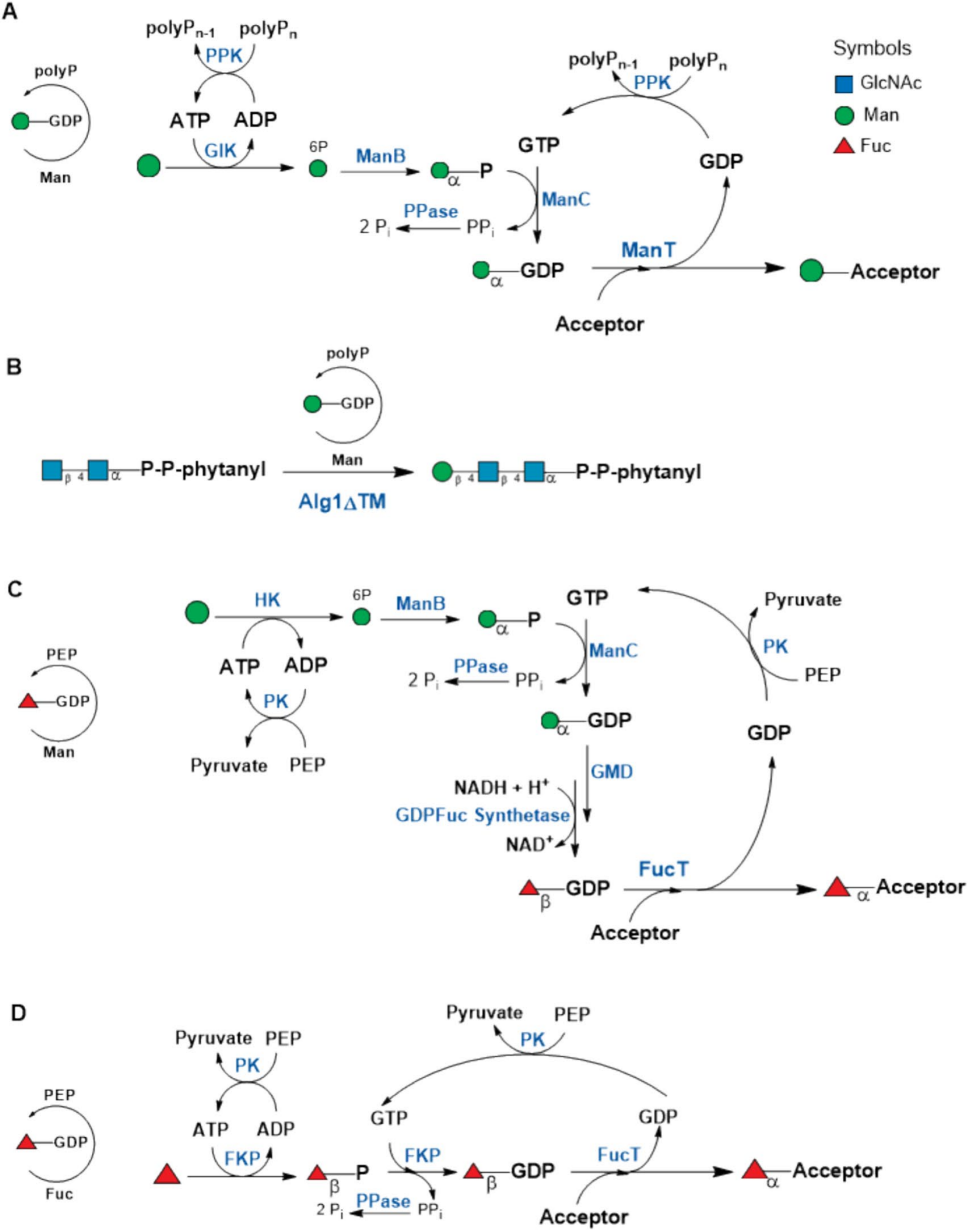
**A** General reaction cascade for GDP-Man regeneration and **B** synthesis of the N-glycan core structure (Rexer et al. 2018). GlK: glucokinase; ManB: phosphomannomutase/PMM; ManC: GDP-Man pyrophosphorylase; ManT: mannosyltransferase (see also Table S1; Supporting Information). **C** Regeneration of GDP-Fuc with de novo pathway enzymes and **D** with FKP from *Bacteriodes fragilis*. NADH regeneration from NAD.^+^ by an alcohol dehydrogenase can be further included (not shown). GMD: GDP-Man 4,6 dehydratase; GDP-Fuc synthetase; FKP: fucokinase/GDP-fucose pyrophosphorylase; FucT: fucosyltransferase (see also Table S1; Supporting Information)

Mannosyl-oligosacharides and -peptides were synthesized with recombinant α1,2-mannosyltransferase (ManT, from yeast). GDP-Man was regenerated in situ starting from Man-1-P which is converted by GDP-Man pyrophosphorylase (ManC, from lyophilized yeast) (Figure S2) (Wang et al. 1993). An extended version for in situ GDP-Man regeneration was established and utilized for the synthesis of the N-glycan core structure (phytanyl-PP-(GlcNAc)-Man_1_) by the β1,4-mannosyltransferase Alg1ΔTM (Fig. 7A) (Rexer et al. 2018). Mannose was first phosphorylated by glucokinase (*Ec*GlcK, Man-6-P) and further converted to Man-1-P (*Ec*ManB) and GDP-Man (*Ec*ManC). ADP and GDP were recycled to ATP and GTP by PPK from *Pseudomonas aeruginosa* (*Pa*PPK) and polyP_n_. Pyrophosphate was hydrolyzed by pyrophosphatase from *Pasteurella multocida* (*Pm*PpA) (Fig. 7B). All enzymes were produced recombinantly in *E. coli*.

For the in situ regeneration of GDP-Fuc de novo pathway enzymes as well as salvage pathway enzymes were employed and started either from d-Man or l-fucose-1-P (Fig. 7). The enzymes for the de novo synthesis of GDP-Fuc derived from permeabilized yeast cells (ManC, GDP-Man synthesis) and a purified enzyme preparation from *Klebsiella pneumoniae* ATCC 12658 (Ichikawa et al. 1992b). Later, the elucidation of the *E. coli* biosynthetic pathway of colanic acid (Andrianopoulos et al. 1998; Stevenson et al. 1996) was a breakthrough for the utilization of the recombinant enzymes GMD and GDP-Fuc synthetase (Fig. 7C). The salvage pathway for GDP-Fuc from Fuc-1-P was accomplished by an enzyme preparation from porcine thyroid glands which contained the GDP-Fuc pyrophosphorylase (Ichikawa et al. 1992b). The observation that the bifunctional enzyme fucokinase/GDP-fucose pyrophosphorylase (FKP) is responsible for the synthesis of GDP-Fuc from l-fucose in *Bacteroides fragilis* (Coyne et al. 2005) paved the way for an effective GDP-Fuc regeneration (Fig. 7D) (Tsai et al. 2013).

#### CMP-Neu5Ac

**Fig. 8:**
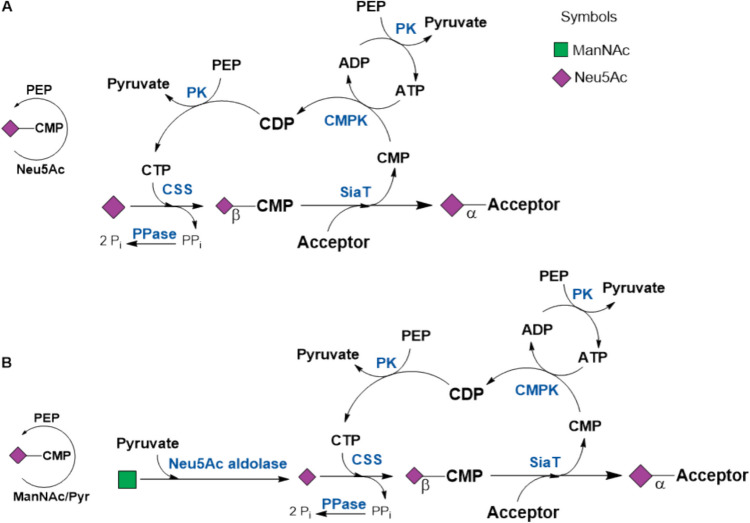
Regeneration of CMP-Neu5Ac. **A** Neu5Ac (Ichikawa et al. 1992b) with PEP/PK, **B** ManNAc/Pyruvate with PEP/PK (Ichikawa et al. 1991; Liu et al. 1992). CSS: CMP-Neu5Ac synthetase from calf brain or recombinant enzyme from *E. coli*; CMPK/CMK: CMP kinase; PPK: polyphosphate kinase, SiaT: sialyltransferase (see also Table S1; SI)

The synthesis of sialylated glycoconjugates afforded the development of regeneration cycles for CMP-Neu5Ac and GDP-Fuc (Ichikawa et al. 1992b). CMP-Neu5Ac was regenerated directly from *N*-acetyl-neuraminic acid (Neu5Ac) (Fig. 8A) or *N*-acetyl-mannosamine (ManNAc) (Fig. 8B, Figure S3) (Ichikawa et al. 1991; Liu et al. 1992; Nahálka and Pätoprstý 2009) . The substrate ManNAc for Neu5Ac aldolase is provided by alkaline treatment or employing a GlcNAc 2’-epimerase (Kragl et al. 1991). NeuAc aldolase utilizes the generated pyruvate as a side product from the pyruvate kinase reaction (Ichikawa et al. 1992a).

#### Nucleotide sugar regeneration cycles in sequential and one-pot reactions

The most interesting study was the multi-gram-scale synthesis of cancer-associated antigens Globo H and SSEA4 (Tsai et al. 2013). Glycosyltransferase reactions were combined with effective regeneration of UDP-Gal, UDP-GalNAc, GDP-Fuc, and CMP-Neu5Ac (Figure S4). For the first time, the efficiency of the regeneration cycles was analyzed by determining the total turnover number for the nucleotides/nucleotide sugars (TTN: moles of product produced per mole of nucleotide present in the reaction solution). UDP-Gal and UDP-GalNAc regeneration yielded TTNs of 80 and 200 for UTP and ATP, respectively. GDP-Fuc regeneration for the synthesis of allyl-Globo H reached 200- and 20-times ATP and GTP recycling, respectively. The synthesis of allyl-SSEA4 was accomplished with in situ regeneration of CMP-Neu5Ac featuring a TTN of 40 for CTP. However, the reaction times for the single steps were between 4 and 10 days. Overall, the cancer-associated antigens Globo H and SSEA4 were obtained in 94% yield (84.46 g) and 54% (2.9 g), respectively.

The one-pot combination of regeneration cycles was demonstrated for the synthesis of 6’-sialyl-LacNAc (Ichikawa et al. 1991), sialyl-Lewis^x^ (Ichikawa et al. 1992b, 1992a) (Figure S5A) as well as for the in situ glycosylation of human IgG (Chung et al. 2006) and for the synthesis of human milk oligosaccharides (Lin et al. 2024). The latter used acetyl-phosphate and acetate kinase for the regeneration of nucleotides. In situ regeneration of UDP-Gal and UDP-GlcNAc was also applied for the sequential synthesis of oligo-LacNAc reaching TTNs of 17 and 44 (UDP-Gal) and 8 and 11 (UDP-GlcNAc) for UTP and ATP, respectively (Wu et al. 2019) (Figure S5B). This was further exploited for the synthesis of bi- and tri-antennary N-glycans with oligo-LacNAc units and their fucosylation and sialylation with in situ regeneration of GDP-Fuc and CMP-Neu5Ac (synthesis of α1,3 fucosides and α2,3, α2,6, and α2,8 sialosides (Anwar et al. 2022) (Figure S5C). As a general strategy, 0.01 eq. ATP and 0.02 eq. NTP for UDP-sugar and CMP-Neu5Ac or 0.02 eq. ATP and 0.04 eq. GTP for GDP-Fuc regeneration was applied in the one-pot synthesis reaction mixtures.

A most recent study reports on the large-scale (5 L) synthesis of LacNAc type 2 (138.6 g isolated product) (Jiao et al. 2024). Key factors for in situ regeneration of UDP-Gal (Fig. 4D) were the concentrations of polyphosphate and Mg^2+^ to avoid phosphate precipitation and to reach highest enzyme activities. However, starting with 10 mM ATP and UTP at substrate concentrations of 100 mM GlcNAc and Gal, respectively, 90.5% LacNAc yield (156.8 g) was reached translating into nine regeneration cycles for ATP and UDP-Gal, respectively. The TTN (g _product_/g _enzyme_) resulted in 37.6-g LacNAc/g β4GalT (2-mg LacNAc/U β4GalT) and a space–time-yield of 1.57 g/L*h.

### Reversible glycosyltransferase reactions for NDP-sugar regeneration

#### Sucrose synthase for NDP-sugar (re)-generation

**Fig. 9:**
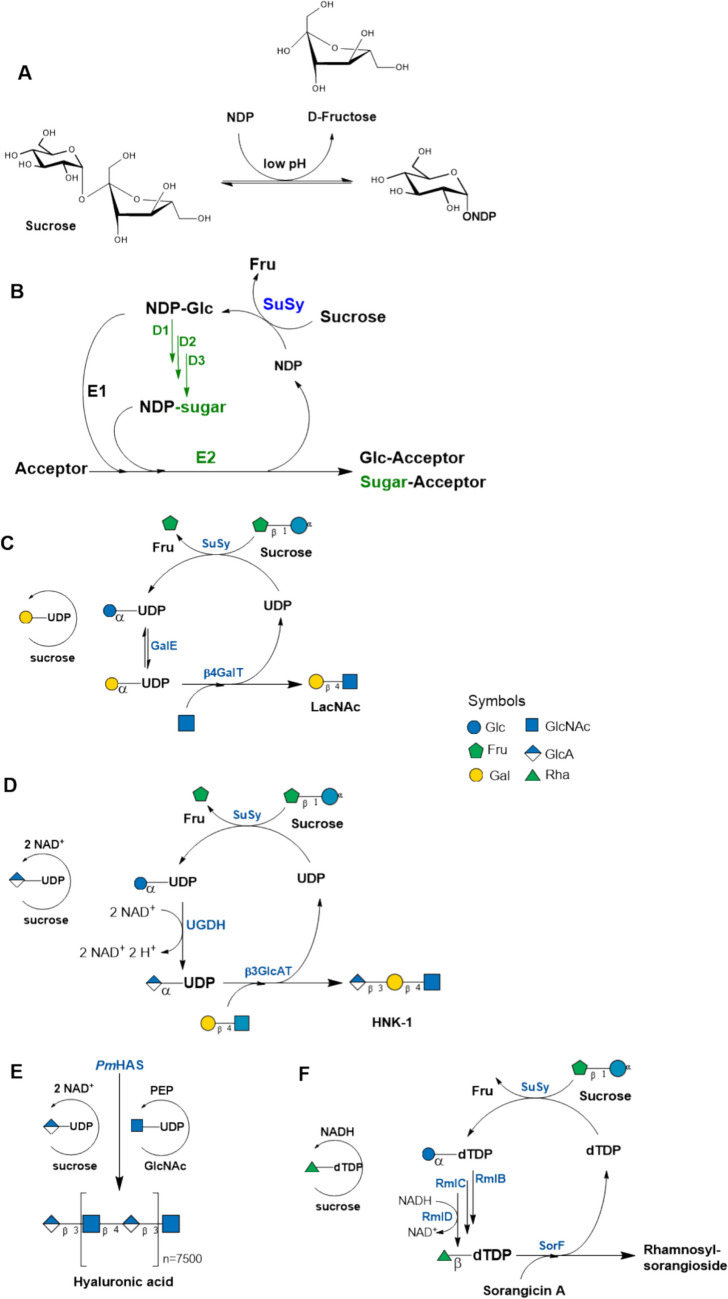
**A** Sucrose synthase (SuSy) reaction and **B** SuSy cycle for the in situ regeneration of NDP-Glc and NDP-sugars (green). D1, D2, D3: NDP-sugar modifying enzymes; E1: GTs utilizing NDP-Glc, E2: GTs utilizing NDP-sugars. In situ regeneration of NDP-sugars from sucrose with sucrose synthase (SuSy). **C** Synthesis of LacNAc with in situ regeneration of UDP-Gal from sucrose and UDP. **D** Synthesis of HNK-1 epitope with in situ regeneration of UDP-GlcA from sucrose and UDP; **E** One-pot synthesis of hyaluronan with regeneration of UDP-GlcA and UDP-GlcNAc; **F** Synthesis of sorangiosides with in situ regeneration of dTDP-d-Glc and dTDP-l-Rha from sucrose and dTDP (see also Table S1; SI)

In our previous studies, we established a novel path for the synthesis of nucleotide sugar from the renewable resource sucrose by the application of sucrose synthase (SuSy, EC 2.4.1.13) (Elling 1997; Schmölzer et al. 2016). SuSy is a unique Leloir glycosyltransferase catalyzing the reversible reaction in vitro and in vivo. The Gibbs free energy of sucrose hydrolysis (ΔG° =  − 29.3 kJ mol^−1^) (Flamholz et al. 2012) is higher compared to that of UDP-Glc (ΔG° =  − 17.6 kJ mol^−1^) and enables the cleavage reaction at low pH values (Fig. 9A). SuSy is a versatile enzyme for the synthesis and in situ regeneration of NDP-Glc and NDP-sugars from sucrose and NDP (Frohnmeyer and Elling 2023) (Fig. 9B).

We applied SuSys from rice (*Oryza sativa*, *Os*Susy) and potato (*Solanum tuberosum*, *St*SuSy1) for the synthesis of UDP-sugars (Engels et al. 2015; Bülter et al. 1997; Bülter and Elling 2000) , dTDP/dUDP-sugars (Elling et al. 2005; Stein et al. 1998, 1995; Zervosen et al. 1996, 1994) as well for ADP-Glc (Zervosen et al. 1998), and CDP-Glc (Zervosen et al. 1996). As a blueprint for the following studies, we demonstrated the first application of SuSy for the in situ regeneration of UDP-Gal in the synthesis of LacNAc (Fig. 9C, Table 1, entry 1) (Elling et al. 1993; Zervosen and Elling 1996) . Compared to the six-enzyme regeneration cycle from Wong et al. (1982) (Fig. 4), only three enzymes are necessary for LacNAc synthesis. To optimize enzyme productivity, the LacNAc synthesis was performed in a repetitive batch mode. Compared to parallel batches, 6–10 times higher mass based turnover numbers (TTNs) (g _product_/g _enzyme_) were reached, however, with an average yield of 57.4% for the synthesis of 0.594-g LacNAc. The SuSy regeneration cycle for UDP-Gal was then further exploited for the synthesis of the Galili epitope (Hokke et al. 1996). The SuSy/GalE cycle was transferred to whole cells (Table 1, entry 2). *E. coli* cells with SuSy from *Anabaena* sp. PCC 7119, *Ec*GalE and α1,4GalT (*Nm*LgtC), and bovine α1,3GalT (*Bos taurus*) to produce globotriose (Gb3) up to 44 mM (22 g/L), as well as the Galili-epitope (α3-Gal) with up to 36 mM (18 g/L) (Chen et al. 2001). In *Pichia pastoris*, 24 mM Galα1,3Lac (Galili-epitope) was synthesized (Shao et al. 2003). Starting from GlcNAc with two UDP-Gal regeneration cycles, 50 mM of P1 trisaccharide (Galα1,4Galβ1,4GlcNAc) (5.4 g in a 200-ml reaction volume) with 67% yield was produced by *E. coli* cells (Liu et al. 2003).

**Table 1:** Application of SuSy regeneration cycles in the synthesis of glycoconjugates and glycosides of secondary metabolites and natural products (see list for abbreviations in Supplementary Information)

Entry	SuSy cycle	Nucleotide sugar	GT	Product	RC^a^	References
**Glycoconjugates **						
1	*Os*SuSy, GalE	UDP-Gal	*Hs*β4GalT1; *Mm*α3GalT	LacNAc, Galili	10	(Elling et al. 1993; Zervosen and Elling 1996; Hokke et al. 1996)
2	*As*SuSy, GalE whole cell	UDP-Gal	*Nm*α4GalT, *Bt*α3GalT, *Hp*β4GalT	Gb3, Galili (α3-Gal), P1 trisaccharide	-^b^	(Chen et al. 2001; Shao et al. 2003; Liu et al. 2003)
3	*St*SuSy1, UGDH, NOX	UDP-GlcA	*Hs*β3 GlcAT-P or *Pm*HAS	Non-sulfated HNK-1 epitope or hyaluronan	24	(Engels et al. 2015; Eisele et al. 2018)
**Glycosides of secondary metabolites and natural products**						
4	*St*SuSy1, RmlB, RmlC, RmlD, FDH	dTDP-d-Glc, dTDP-4-keto-6-deoxy-Glc dTDP-l-Rha	*Sc*SorF	Sorangiosides	10	(Rupprath et al. 2007)
5	*At*SuSy1	UDP-Glc	*Cr*UGT2, F7GT, *Cr*UGT73A15, *Bs*YjiC	Curcumin glucosides, apigenin glucosides; quercitin and resveratrol glucosides, pterostilbene O-glucoside	17	(Masada et al. 2007; Terasaka et al. 2012; Dai et al. 2017)
6	*At*SuSy1	UDP-Glc	*Os*UGT79	Deoxynivalenol-3-O-β-d-glucoside (D3G)	100	(Michlmayr et al. 2015)
7	*At*SuSy1	UDP-Glc	*Bs*YjiC	Rebaudioside L2	-^b^	(Yang et al. 2023)
8	*At*SuSy1	UDP-Glc	*At*UGT73C5, *Ca*UGT3	Cinnamyl alcohol mono-glucoside (rosin), rosavin B	-^b^	(Han et al. 2023)
9	*Gm*SuSy	UDP-Glc	*Os*CGT, *Pc*OGT	Nothofagin (C-glucoside), davidioside and confusoside	44–220	(Bungaruang et al. 2013; 2016; Schmölzer et al. 2018)
10	*Gm*SuSy	UDP-Glc	*Ct*UGT71E5	15-hydroxy-cinmethylin-glucoside, toxin	9	(Jung et al. 2021)
11	*GmSuSy*	UDP-Glc	Microbial UGT_BL_1	Cinnamyl alcohol mono-glucoside (rosin)	36	(Chu et al. 2024)
12	*St*SUS1	UDP-Glc	*Sl*UGTSL2	Steviol glycosides, rebaudioside D	310^c^	(Chen et al. 2018)
13	*St*SUS1	UDP-Glc	*Ac*UGT73G1	Quercetin-3,4′-O-diglucoside	-^b^	(Sun et al. 2020)
14	*Gu*SUS1 variant, GuSUS1-Δ9	UDP-Glc	UGT73C11	Glycyrrhetinic acid 3-O-glucoside	4	(Liu et al. 2024)
15	*Mc*SuSy S31D variant		*At*UGT78D2*, Ac*UGT73G1; *Sr*UGT76G1/S195Q	Quercetin-3,4 ‘-*O*-di-glucoside; rebaudioside D/M	-^b^	(Tao et al. 2023; Chen et al. 2023)
16	*Ac*SuSy*; Ac*SuSy- L637M/T640V variant	UDP-Glc	*Os*CGT	Nothofagin (C-glucoside)	-^d^	(Gutmann et al. 2017; Diricks et al. 2017)
17	*Gm*SuSy co-immobilized with UGT	UDP-Glc	*Bl*YjiC; OleD, *Os*CGT	Sissotrin, flavonoid glucosides, nothofagin	8.0, 10.0	(Matera et al. 2022, 2024; Liu et al. 2022)
18	*Gm*SuSy *P*. *pastoris* surface	UDP-Glc	*Gu*UGT73F24m-P- pastoris surface	Glycyrrhetinic acid 3-O-glucoside	-^d^	(Guo et al. 2023)
19	*Ac*SuSy or *Ac*SuSy variant L637M/T640V, co-immobilized with UDP and UGT	UDP-Glc	*Md*UGT71A15,	Flavonoid glucosides	50^e^	(Trobo-Maseda et al. 2023)
20	*At*SuSym, SpyT-SpyC fusion, immobilized, magnetic nanoparticles	UDP-Glc	*Bs*UGT2m from *Bacillus subtilis*	Curcumin glucoside	-^d^	(Ali et al. 2024)
21	*At*SuSy1, fusion with UGT	UDP-Glc	*Stevia* UGT76G1	Rebaudioside A,	-^d^	(Tao et al. 2022)
22	*At*SuSy1, *E. coli* whole cell	UDP-Glc	OleD; plant UGTs	Rebaudioside KA; lignan glucosides	-^b^	(Zhang et al. 2020; Qiao et al. 2024)
23	*Gm*SuSy, *E. coli*, in vitro, and whole cell	UDP-Glc	*It*UGT2	Gastrodin, *p*-hydroxybenzoic acid glucoside	37	(Cui et al. 2023)
24	mbSUS whole cell *P. pastoris*	UDP-Glc	*Sr*UGT76G1	Rebaudioside A	-^d^	(Chen et al. 2021)
25	*Gm*SuSy, GalE	UDP-Gal	*Ph*UGT	Hyperoside O-glycoside	18	(Pei et al. 2017)
26	*Gm*SuSy, *Vv*RHM-*At*NRS/ER	UDP-l-Rha	*At*UGT78D1	Quercetin 3-O-rhamnoside (quercitrin)	-^d^	(Pei et al. 2018)
27	*At*SuSy1*Gm*SuSy, *At*RHM	UDP-l-Rha	*At*UGT89C1	Quercetin 7-O-rhamnoside	10	(Thapa et al. 2019)
28	*Gu*SuS1-Δ9, *At*UGDH3, *At*UX3, *Ps*UGE2	UDP-l-Ara	*As*UGT99D1	Betulinic acid 3-O-arabinoside	-^d^	(Sun et al. 2023)

^a^*RC* Regeneration cycles for NDP-sugar; ^b^UDP concentration unknown in crude extract; overestimated with additional UDP in crude extract; ^d^not reported; ^e^accumulated TTN (mol product mol UDP^−1^) after 5 reaction cycles

Including a UDP-Glc dehydrogenase (UGDH), a SuSy cycle was created for the in situ regeneration of UDP-GlcA and applied in the synthesis of the non-sulfated HNK-1 epitope (Fig. 9D, Table 1, entry 3) (Engels et al. 2015). The biopolymer hyaluronic acid with an average molecular weight of 3 MDa was synthesized with hyaluronan synthase from *Pasteurella multocida* (*Pm*HAS) (Fig. 9E, Table 1, entry 3) (Eisele et al. 2018). The regeneration cycle included UDP-glucose dehydrogenase from *Streptococcus zooepidemicus* (*Sz*UGDH). NAD^+^ was regenerated from NADH + H^+^ by NADH oxidase from *Lactobacillus brevis* (*Lb*NOX) (not shown).

The promiscuity of *St*SuSy1 for different nucleoside diphosphates opened the path to the glycosylation of natural products employing the SuSy regeneration cycle. We demonstrated for the first time the in situ regeneration of dTDP-d-Glc and dTDP-l-Rha in the synthesis of sorangiosides (Fig. 9F, Table 1, entry 4) (Rupprath et al. 2007).

#### SuSy regeneration cycles in the synthesis of glycosylated secondary metabolites

In recent years, the SuSy cycle for the in situ regeneration of UDP-Glc was applied in combination with different plant UDP-sugar glycosyltransferases (UGTs) for the glucosylation of plant secondary metabolites. Table 1 summarizes reports from which only those are discussed with impact on the created regeneration cycles. To reach high regeneration cycle numbers, suitable SuSy enzymes or engineered variants thereof with high affinity for UDP were applied.

SuSys from plants like *Solanum tuberosum* (*St*SuSy1), *Glycine max* (*Gm*SuSy), and *Arabidopsis thaliana* (*At*SuSy) display a kinetic preference for UDP (Römer et al. 2004; Gutmann et al. 2014, 2017; Terasaka et al. 2012). Most of the plant SuSy enzymes were recombinantly produced in *E. coli*, apart from *St*SuSy1 which was produced in *Saccharomyces cerevisiae*. For *St*SuSy1, a K_M_ of 4 µM and 91.6 mM was reported for UDP and sucrose, respectively (Römer et al. 2004). The relatively low K_M_ for UDP was attributed to the phosphorylation at S11 of *St*SuSy1 in the yeast expression system, similar to the native plant enzyme (Sauerzapfe et al. 2008). SuSy enzymes produced in *E. coli* display a *K*_*M*_ value for UDP and sucrose in the range of 0.1–1 mM and 20–100 mM, respectively. To achieve high numbers for regeneration cycles, a low *K*_*M*_ of SuSy enzymes for UDP is beneficial. UDP is also an effective inhibitor of UGTs. As a general strategy for effective UDP-Glc regeneration, a system *K*_*M*_ for a SuSy-UGT cascade was defined where the UDP concentration is well above the *K*_*M*_ for the applied SuSy and low enough to avoid inhibition of the GT (Gutmann et al. 2017).

*At*SuSy1 was demonstrated to catalyze 100-fold recycling of UDP-Glc (Table 1, entry 6) (Michlmayr et al. 2015). A crude extract of co-expressed *At*SuSy1 and *At*UGT73C5 yielded 94% cinnamyl alcohol mono-glucoside without the addition of UDP (Table 1, entry 8) (Han et al. 2023). SuSy from soybean (*Gm*SuSy, *K*_*M*_ UDP 0.13 mM; *K*_*M*_ sucrose 25.5 mM) (Bungaruang et al. 2013) regenerated UDP-Glc 220-times in a process for the synthesis of the C-glucoside nothofagin (Schmölzer et al. 2018) (Table 1, entry 9).

However, SuSy enzymes from green algae (*Mc*SuSy) and the thermophilic bacterium *Acidithiobacillus caldus* (*Ac*SuSy) were applied for in situ regeneration of UDP-Glc (Table 1, entries 15 and 16). The L637M/T640V variant of *Ac*SuSy with a 60-fold lower *K*_*M*_ value for UDP (0.13 mM) has also been used for the in situ regeneration of UDP-Glc (Table 1, entry 16) (Gutmann et al. 2017; Diricks et al. 2017). The high affinity for UDP was exploited for the co-immobilization of *Ac*SuSy with UDP reaching an RC for UDP of 50 after five reaction cycles without the addition of UDP (Table 1, entry 19) (Trobo-Maseda et al. 2023). Fusion proteins of *At*SuSy and a respective UGT were created for efficient synthesis of curcumin glucoside and rebaudioside A (Table 1, entries 20 and 21). *Gm*SuSy or *At*Susy were co-immobilized (Table 1, entries 17 and 20) or co-displayed with UGTs on the cell surface (Table 1, entry 18), or applied as whole-cell biocatalysts (Table 1, entry 22–24) for UDP-Glc regeneration. Notably, co-immobilization of *Gm*SuSy and *Bl*YjiC on an EnziG Opal resin resulted in excellent reusability over 12 cycles with 96% product yield (Table 1, entry 17) (Matera et al. 2022). Significantly higher product yields were attributed to substrate channeling effects by co-displaying *Gm*SuSy and *Gu*UGT73F24m in proximity on the surface of *Pichia pastoris* (Table 1, entry 18)(Guo et al. 2023).

The SuSy/GalE cycle was exploited for the synthesis of hyperoside O-galactosides reaching an RC number of 18 (Table 1, entry 25) (Pei et al. 2017). Similar to the regeneration of dTDP-l-Rha (Rupprath et al. 2007), SuSy cycles were extended for the in situ regeneration of UDP-l-Rha.

The fusion of *Vv*RHM and *At*NRS/ER gave an NADPH-independent self-sufficient biocatalyst for the synthesis of UDP-l-Rha from UDP-Glc (Table 1, entry 26) (Pei et al. 2018). Notably, an RC number of 10 was reached for the synthesis of quercetin 7-O-α-l-rhamnoside (Table 1, entry 27) (Thapa et al. 2019). A four-enzyme cascade was applied comprising sucrose synthase, UDP-Glc dehydrogenase, UDP-*α*-d-glucuronic acid decarboxylase, and UDP-Glc 4’-epimerase for the generation of UDP-l-Ara from UDP-Glc (Table 1, entry 28) (Sun et al. 2023). Notably, only 6.67 mM sucrose was applied for the reaction of truncated SuSy *Gu*SUS1-Δ9) from licorice (*Glycyrrhiza uralensis*). However, UDP was applied in tenfold excess to the acceptor substrate and in situ regeneration of UDP-l-Ara was not demonstrated.

#### NDP cycling with energy-rich glycosides

The reversible sugar transfer by Leloir glycosyltransferases was also shown in natural product biosynthesis (Minami et al. 2005; Bode and Müller 2007) . In this approach, the NDP-sugar is generated in situ from a natural glycoside and NDP (chemical initiator) by the reverse GT reaction and transferred to a new aglycone by the same GT (aglycone exchange) (Figure S6). The GT VinC from the vicenistatin biosynthesis pathway was demonstrated to transfer dTDP-vicenisamine onto a variety of aglycons, thereby generating a library of new vicenisaminyl-glycosides. Conversion yields of up to 42% were obtained (Minami et al. 2005). This concept was further expanded to exchange the sugar moiety of natural products (Zhang et al. 2006). GTs from calicheamicin and vancomycin biosynthetic pathways catalyze reversible reactions, facilitating the exchange of sugars and aglycons. To simplify the natural sugar substrates, energy-rich aromatic glycosides (2-chloro-4-nitrophenyl glycosides) and NDPs were used for the in situ generation of NDP-activated sugars (Gantt et al. 2011). The single- and dual-GT coupled reactions for sugar transfer were harnessed for the glycodiversification of natural products. The study also introduces a high-throughput colorimetric assay to monitor glycosyltransferase activity in real-time, facilitating rapid screening for novel acceptors and enzyme engineering. In a recent study, an HTS system with *p*NPβ-Glc to generate UDP-Glc from released UDP was developed to screen for aglycone promiscuity of a GT variant library (phenols, phenylpropanoids, and flavonoids) (Zhang et al. 2023). However, critical points for applications in preparative synthesis are the high amounts of enzymes, the chemical preparation of activated glycosides, and the unfavorable equilibria of the GT reactions to achieve economic and high product yields.

#### Reverse glycosylation of GTs with lactose and sialosides

The reversibility of the bacterial β4GalT *Nm*LgtB-B was exploited to synthesize LacNAc type 2 from lactose with in situ regeneration of UDP-Gal (Huang et al. 2019). The reaction yielded 90% conversion. However, an over-stoichiometric concentration of UDP with reference to the acceptor and > 20-fold excess of lactose renders this type of reversible GT reaction as a transgalactosylation from lactose by in situ generation of UDP-Gal from lactose. This type of reaction does not fulfill the requirements for in situ regeneration of nucleotide sugars, and still uses a high amount of the expensive UDP.

Sialyltransferases catalyze the reverse sialylation in the presence of CMP and sialosides generating CMP-Neu5Ac, which is utilized in the transfer reaction of the same or another SiaT. This was demonstrated for the mammalian ST3Gal-II producing CMP-Neu5Ac in situ which is utilized by ST6Gal-I and ST6GalNAc-I to form α2,6-sialylated O-glycans (Chandrasekaran et al. 2008, 2011). Also, bacterial α2,6-SiaTs from the GT families GT80 and GT54 display a reversible reaction in the presence of CMP (Mehr and Withers 2016; McArthur et al. 2018; Both et al. 2018). The in situ formed CMP-Neu5Ac is subsequently hydrolyzed (transfer of Neu5Ac onto water) to yield CMP and Neu5Ac. The reverse sialylation with in situ formation of CMP-Neu5Ac was demonstrated, however, not yet been exploited for preparative synthesis.

#### Critical points

To achieve high regeneration cycles (RC) or total turnover numbers (TTN, mol formed product per mol nucleotide) in the nucleotide sugar regeneration cycles, kinetic characterization of the GTs for their respective nucleotide sugar and nucleotide (for the reverse reaction) is demanding. This applies also to the enzymes converting N(M)DPs to NTPs for the synthesis of nucleotide sugars (e.g., by salvage pathway enzymes). Based on these data, enzyme cascades for nucleotide sugar regeneration with high TTNs shall be optimized and calculated by reaction modeling. Another critical point is the number of enzymes in such regeneration cycles and their repetitive use. Immobilization and protein fusion strategies have been explored, however, being optimized for every single enzyme cascade. In most studies, one-pot synthesis approaches are reported leading to a compromise concerning optimal reaction conditions (T, pH, buffer) for the nucleotide sugar-synthesizing enzymes and GTs. Compartmentalization of enzyme cascades shall be envisaged to decouple enzyme cascades and gain effective regeneration cycles. This principle shall be further followed to integrate in situ nucleotide sugar regeneration cycles in reactors for automated enzymatic glycan synthesis (Hussnaetter et al. 2023).

## Conclusions

This review summarizes the course of the development of in situ nucleotide sugar regeneration cycles. The overarching principle is to create economic synthetic strategies for glycoconjugates employing Leloir GTs and nucleotide sugars. The pioneering work of Wong and Whitesides demonstrated the principle that enzymes taken from biosynthetic pathways for nucleotide sugars and nucleotides can be combined with GTs. Another important milestone was the discovery of salvage pathways for many nucleotide sugars further reducing the number of enzymes in the regeneration cycles. A breakthrough was the demonstration of the reversible sucrose synthase (SuSy) reaction to regenerate NDP-sugars from sucrose. This paved the way for a plethora of studies on the synthesis of glycosylated natural compounds. The principle of the reverse GT reaction to generate nucleotide sugars from glycosides was then also demonstrated for eukaryotic and prokaryotic Leloir GTs. The application of regeneration cycles has been advanced by enzyme immobilization for multiple enzyme cycles and reactor engineering. In summary, in situ regeneration of nucleotide sugars is an advanced field in glycobiotechnology for economic access to high-value products.

## Supplementary Information

Below is the link to the electronic supplementary material.

Supplementary file1 (PDF 545 KB)
