# Supplementary material for: Enzyme cascades for nucleotide sugar regeneration in glycoconjugate synthesis
Source: Appl Microbiol Biotechnol. 2025 Feb 27;109(1):51. doi: 10.1007/s00253-025-13432-2 (PMC11868170; doi:10.1007/s00253-025-13432-2)
Supplement: Supplementary file 1 — Supplementary file1 (PDF 545 KB) [file 253_2025_13432_MOESM1_ESM.pdf]

Supplementary Information

Applied Microbiology and Biotechnology

Enzyme Cascades for Nucleotide Sugar Regeneration in Glycoconjugate Synthesis

Lothar Elling*

Laboratory for Biomaterials, Institute of Biotechnology, and Helmholtz-Institute for Biomedical Engineering, RWTH Aachen University, Pauwelsstraße 20, 52074 Aachen, Germany,

*Corresponding author: e-mail: l.elling@biotec.rwth-aachen.de, telephone: +49 241 8028350, telefax: +49 241 80 22387

Abbreviations:

Sucrose synthases:

*Ac***SuSy**: SuSy from *Acidithiobacillus caldus;At***SuSy1**: SuSy1 from *Arabidopsis thaliana*; *Gm***SuSy**: SuSy from soybean (*Glycine max*); *Gu***SUS1**: SuSy1 from licorice (*Glycyrrhiza uralensis)*; **mbSUS**: SuSy from *Vigna radiata*; *Mc***SuSy**: SuSy from *Micractinium conductrix*; *Os***SuSy**: SuSy from rice (*Oryzae sativa*); *St***SuSy1/***St***SUS1**: SuSy1 from potato (*Solanum tuberosum*);

Enzymes for NDP-sugar recycling:

*At***UGDH3**: UDP-Glc dehydrogenase from *Arabidopsis thaliana; At***USP**: UDP-sugar pyrophosphorylase from *Arabidopsis thaliana*; *At***RHM1**: UDP-rhamnose synthase from *Arabidopsis thaliana*; *At***UX3**: UDP-GlcA decarboxylase from *Arabidopsis thaliana*; *At***GalE:** UDP-Glc 4'-epimerase; *Bf***FKP**: fucokinase/ GDP-fucose pyrophosphorylase from *Bacillus fragilis*; *Bl***NahK**: *N*-acetyl-hexosamine kinase from *Bifidobacterium longum ssp. infantis*; **CMPK/CMK**: CMP kinase; **CSS**: CMP-Neu5Ac synthetase; *Ec***CMK**: Cytidin-5'-monophospahte kinase from *Escherichia coli*; *Ec***GalK**: galactokinase from *Escherichia coli*; *Ec***GlmU**: UDP-GlcNAc/GalNAc pyrophosphorylase from *Escherichia coli; Ec***PK**: Pyruvate kinase from *Escherichia coli*; **FDH**: formate dehydrogenase; **GalU**: UDP-Gal uridylyltransferase; *Lb***NOX**: NADH oxidase from *Lactobacilllus brevis*; **NRS/ER**: UDP-4-keto-6-deoxy-D-glucose 3,5-epimerase/UDP-4-keto-rhamnose 4-keto-reductase; *Ps***UGE2**: UDP-Glc 4'-epimerase from *Pisum sativum*; **PGM**: phosphoglucomutase; **PK**: pyruvate kinase; *Pm***CSS**: CMP-Neu5Ac synthetase from *Pasteurella multocida*; **PPK**: polyP$_n$ kinase; **RmlB**: dTDP-Glc 4,6-dehydratase; **RmlC**: dTDP-4-keto-6-deoxy-Glc 3,5-epimerase, **RmlD**: dTDP-4-keto-6-deoxy-L-mannose 4-ketoreductase; **UGDH**: UDP-Glc dehydrogenase; **UDP-Glc PP***:* UDP-Glc pyrophosphorylase; **USP**: UDP-sugar pyrophosphorylase; *Vv***RHM**: UDP-rhamnose synthase from *Vitis vinifera.*

Leloir-Glycosyltransferases:

*As***UGT99D1**: UDP-arabinosyltransferase from *Avena strigose*; *At***UGT73C5**: UDP-glucosyltransferase from *Arabidopsis thaliana*; *At***UGT78D1**: UDP-rhamnose flavonol glycosyltransferase from *Arabidopsis thaliana*; *At***UGT78D2**: UDP-glucosyltransferase from *Arabidopsis thaliana*; *As***UGT99D1**: UDP-arabinosyltransferase from *Avena strigose; Bs***YjiC**: UDP-glucosyltransferase from *Bacillus subtilis* 168; *Ca***UGT3**: 1,6-glucosyltransferase from *Catharanthus roseus*; *Ca***UGT73A15**: UDP-glucosyltransferase from *Catharanthus roseus*; *Ct***UGT71E5**: UDP-glucosyltransferase from safflower (*Carthamus tinctorius*); *Cr***UGT2**: UDP-glucosyltransferase from *Catharanthus roseus*; **F7GT**: flavonoid 7-O-glucosyltransferase from *S. baicalensis*; *Gu***UGT73C11**: UDP-sugar glycosyltransferase from *Glycyrrhiza uralensis*; *Gu***UGT73F24m**: I23G/L84N mutant UDP-glucosyltransferase from *Glycyrrhiza uralensis*; *Hi***LgtD**: β1,3GalNAcT from *Haemophilus influenzae*; *Hp***FutC**: α1,2Fucosyltransferase from *Helicobacter pylori*; *Hs***β3GlcAT-P**: β3-glucuronosyltransferase P from *Homo sapiens*; *Hs***β4GalT1**: β4-galactosyltransferase 1 from *Homo sapiens*; *It***UGT2**: UDP-glucosyltransferase from *Indigofera tinctorial*; *Mm***α3GalT**: α3GalT from mouse (*Mus musculus*); *Md***UGT71A15**: UGT from *Malus domestica*; *Nm***LgtC**: α1,4Galactosyltransferase from *Neisseria meningitidis*; **OleD**: UDP-glucosyltransferase from *Streptomyces antibioticus*; *Os***CGT**: C-glucosyltransferase from rice (*Oryza sativa*); *Os***UGT79**: UDP-glucosyltransferase from rice (*Oryza sativa); Pc***OGT**: UDP-glucosyltransferase from pear (*Pyrus communis*); *Sc***Sorf**: Sorf from *Sorangium cellulosum*; *Sr***UGT76G1/S195Q**: UDP-glucosyltransferase from *S. rebaudiana*; *Sr***UGT76G1** :UDP-sugar glycosyl transferase from *Stevia rebaudiana Bertoni*; **Sia T**: sialyltransferase; **UGTs**: UDP-glucosyltransferases from plants; **UGT$_{BL}$1**: Microbial glycosyltransferase; **VspJT-FAJ-16**: α2,3Sialyltransferase; **UGTSL2**: UDP-sugar glycosyltransferase from *Solanum lycopersicum.*

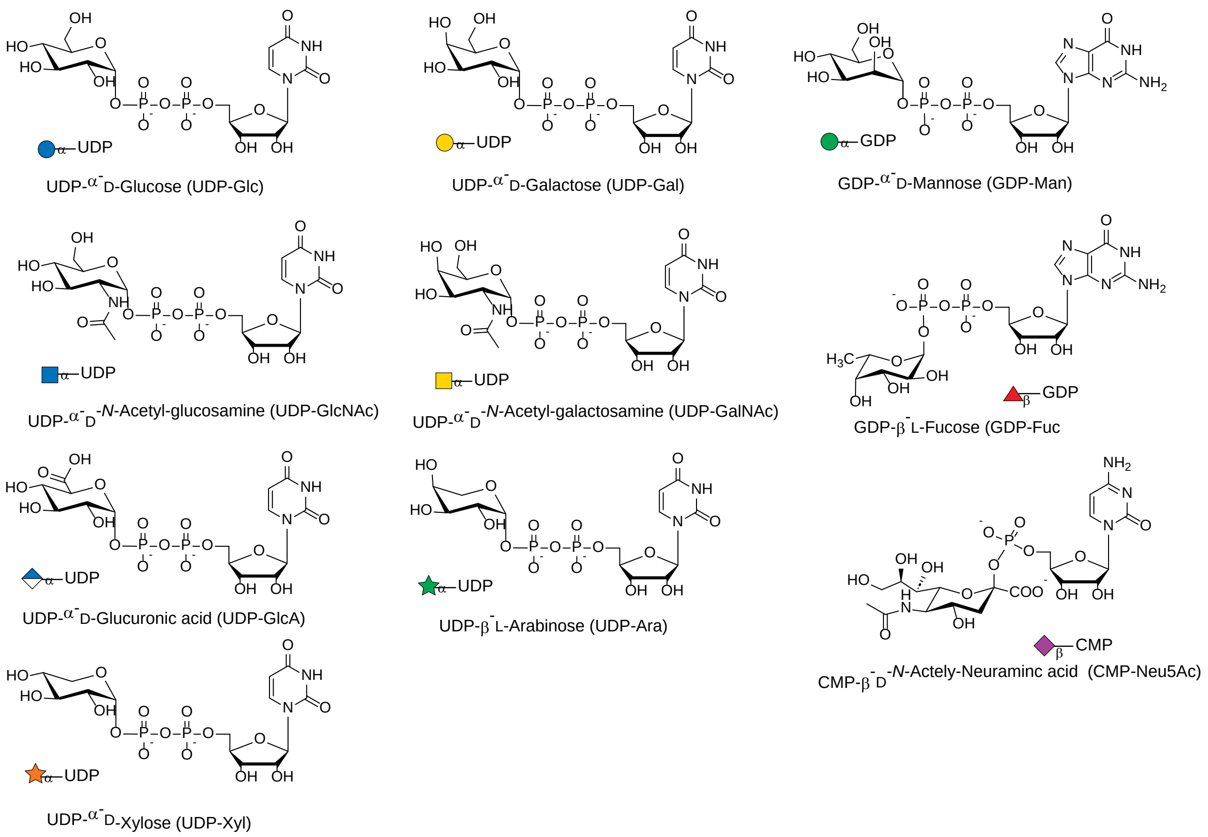

SHAPE	White (Generic)	Blue	Green	Yellow	Orange	Pink	Purple	Light Blue	Brown	Red
Filled Circle	Hexose	Glc	Man	Gal	Gul	Alt	All	Tal	Ido	
Filled Square	HexNAc	GlcNAc	ManNAc	GalNAc	GulNAc	AltNAc	AllNAc	TalNAc	IdoNAc	
Crossed Square	Hexosamine	GlcN	ManN	GalN	GulN	AltN	AllN	TalN	IdoN	
Divided Diamond	Hexuronate	GlcA	ManA	GalA	GulA	AltA	AllA	TalA	IdoA	
Filled Triangle	Deoxyhexose	Qui	Rha		6dGul	6dAlt		6dTal		Fuc
Divided Triangle	DeoxyhexNAc	QuiNAc	RhaNAc			6dAltNAc		6dTalNAc		FucNAc
Flat Rectangle	Di-deoxyhexose	Oli	Tyv		Abe	Par	Dig	Col		
Filled Star	Pentose		Ara	Lyx	Xyl	Rib				
Filled Diamond	3-deoxy-nonulosonic acids		Kdn				Neu5Ac	Neu5Gc	Neu	Sia
Flat Diamond	3,9-dideoxy-nonulosonic acids		Pse	Leg		Aci		4eLeg		
Flat Hexagon	Unknown	Bac	LDmanHep	Kdo	Dha	DDmanHep	MurNAc	MurNGc	Mur	
Pentagon	Assigned	Api	Fru	Tag	Sor	Psi				

Scheme S1 Chemical Structures, symbol presentation, names, and abbreviations of common nucleotide sugars (Frohnmeyer, Elling 2023). Common nucleotide sugars are depicted according to the symbol nomenclature for graphical representations of glycans (Varki et al. 2015).

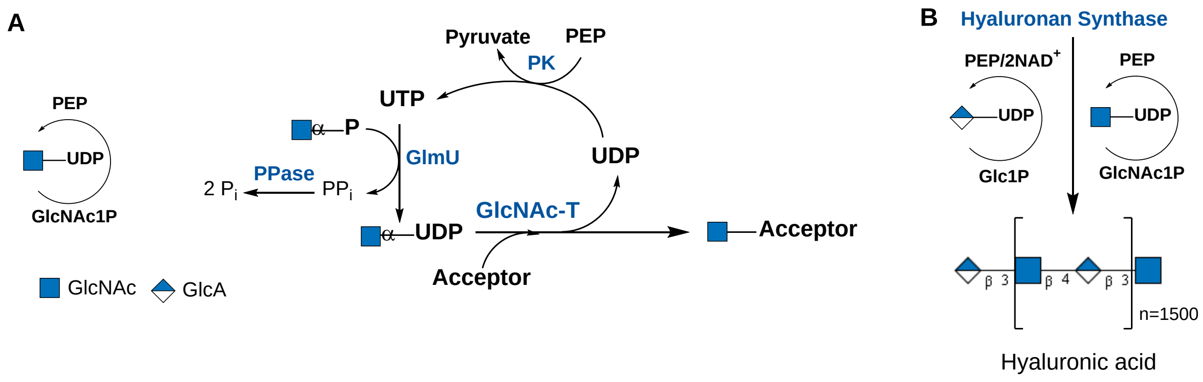

Figure S1 A Regeneration cycle for UDP-GlcNAc and synthesis of hyaluronic acid with *in situ* regeneration of UDP-GlcA and UDP-GlcNAc (De Luca et al. 1995).

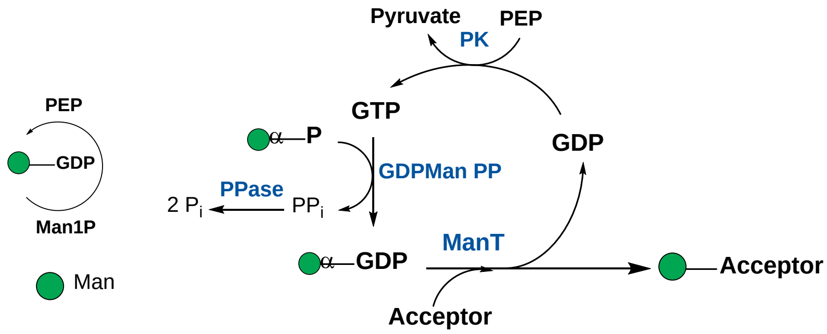

Figure S2 Regeneration cycle for GDP-Man (Wang et al. 1993).

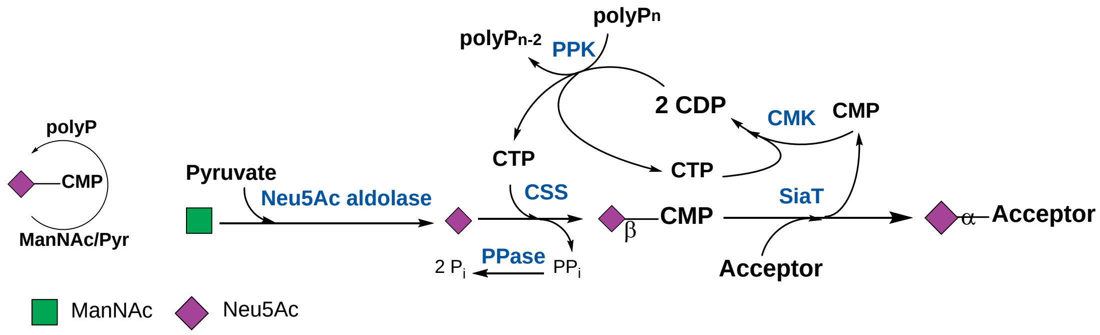

Figure S3 Regeneration of CMP-Neu5Ac from ManNAc/pyruvate with polyP/PPK (Nahálka, Pätoprstý 2009). CSS: CMP-Neu5Ac synthetase from calf brain or recombinant enzyme from *E. coli*; CMPK/CMK: CMP kinase; PPK: polyphosphate kinase, SiaT: sialyltransferase.

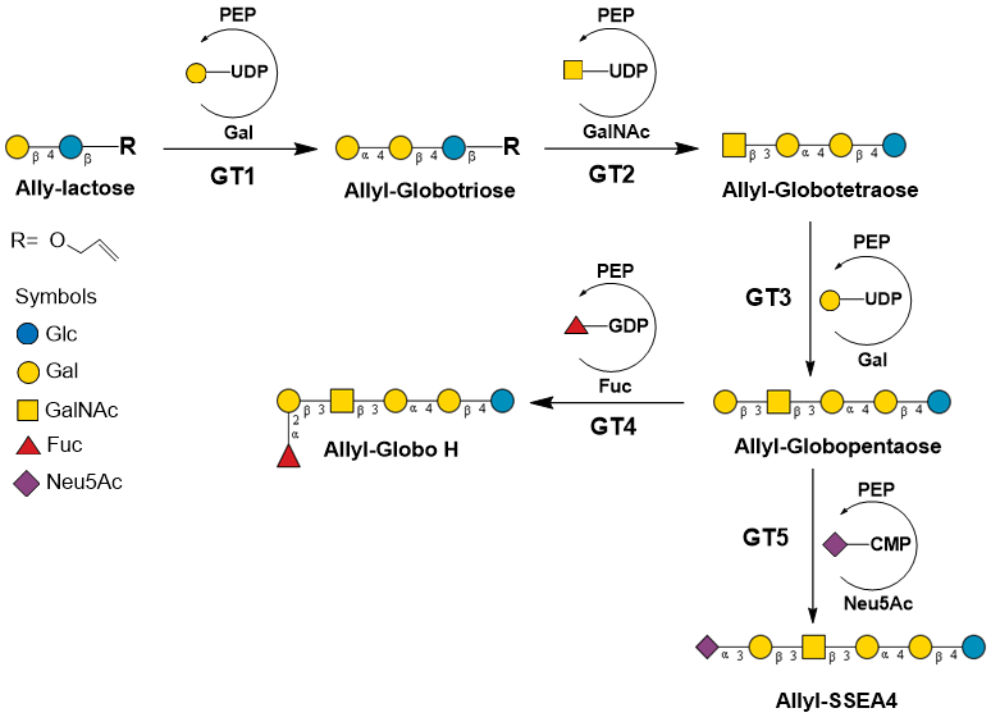

Figure S4 Multi-gram scale sequential synthesis of the tumor-associated antigens Globo H and SSEA4 with *in situ* nucleotide sugar regeneration (Tsai et al. 2013). UDP-Gal cycle with *Ec*GalK, *At*USP; UDP-GalNAc cycle with *Bl*NahK, *Ec*GlmU; GDP-Fuc cycle with *Bf*FKP; CMP-Neu5Ac cycle with *Ec*CMK, *Pm*CSS; *Ec*PK was used for nucleotide regeneration. GT1: α1,4GalT (*Nm*LgtC); GT2/GT3: β1,3GalNAcT (*Hi*LgtD); GT4: α1,2FucT (*Hp*FutC); GT5: α2,3SiaT (*Vsp*JT-FAJ-16) (see also **Table S1**; SI).

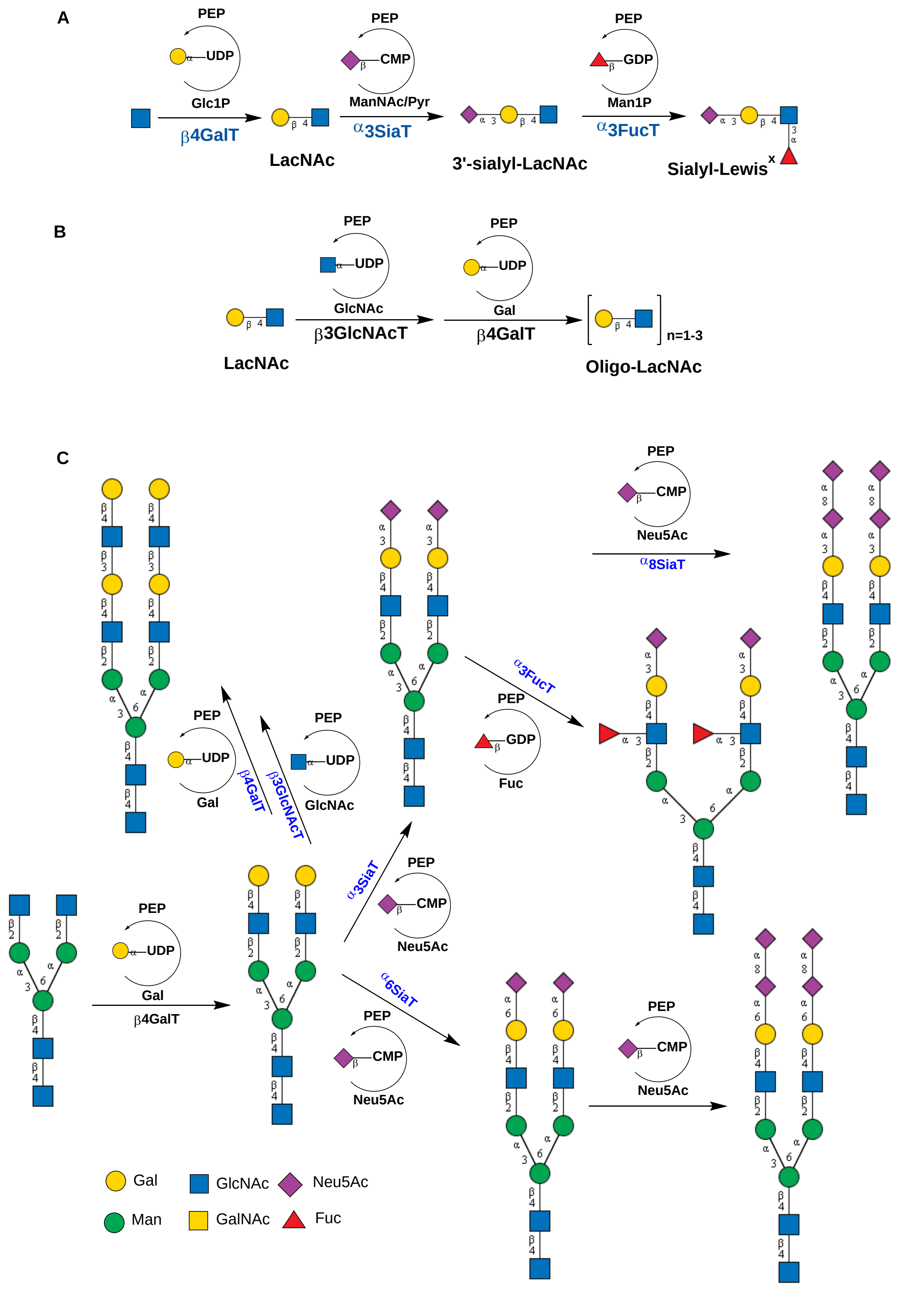

Figure S5 **A** One-pot synthesis of sialyl-Lewis^x with *in situ* regeneration of UDP-Gal, CMP-Neu5Ac and GDP-Fuc (Ichikawa et al. 1992b; Ichikawa et al. 1992a). **B** Sequential synthesis of oligo-LacNAc with *in situ* regeneration of UDP-GlcNAc and UDP-Gal (Wu et al. 2019). **C** Sequential synthesis of sialylated and fucosylated bi-antennary N-glycans (Anwar et al. 2022).

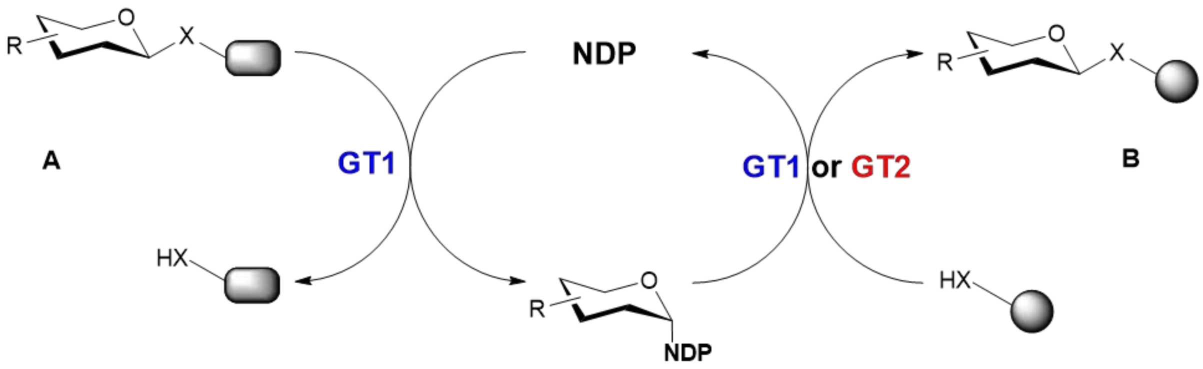

Figure S6 **A** Single GT-coupled and **B** dual GT-coupled aglycone exchange by reverse GT reactions (Minami et al. 2005; Gantt et al. 2011).

Table S1 Enzyme cascades for the *in situ* regeneration of nucleotide sugars

Regeneration cycle	Number of enzymes for regeneration of	Stoichiometric substrates or in excess	References
		**UDP-Glc**	
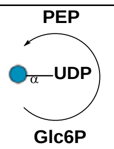	**4**: PGM, UDP-Glc PP; PPase, PK	Glc6P, PEP	(Haynie, Whitesides 1990)
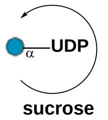	**1**: SuSy	Sucrose	(Masada et al. 2007)
		**UDP-Gal**	
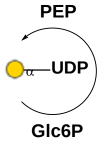	**5:** PGM, UDP-Glc PP; PPase, GalE, PK	Glc6P, PEP	(Wong et al. 1992)
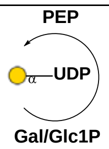	**5:** GalK, UDP-Glc PP, PPase, GalU, PK	**Gal**, Glc1P, PEP	(Wong et al. 1992)
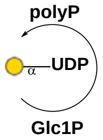	**4:** UDP-Glc PP; PPase, GalE, PPK	Glc1P, polyP	(Noguchi, Shiba 1998)
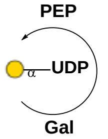	**4**: GalK, USP, PPase, PK	Gal, PEP	(Tsai et al. 2013; Wu et al. 2019)
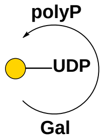	**4**: GalK, USP, PPase, PPK	Gal, polyP	(Jiao et al. 2024)
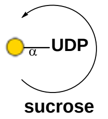	**2**: SuSy, GalE	Sucrose	(Zervosen, Elling 1996; Hokke et al. 1996; Pei et al. 2017)
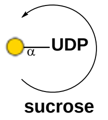	**3**: SuSy, GalU	Sucrose, Gal1P	(Zervosen, Elling 1996)

Table S1 continued

**Regeneration cycle**	**Number of enzymes for regeneration of**	**Stoichiometric substrates or in excess**	**References**
**UDP-GlcA**			
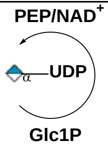	**4:** UDP-Glc PP; PPase, UGDH, PK **5:** 4+ enzyme for NAD^+ regeneration	Glc1P, PEP, (NAD^+)	(Gygax et al. 1991)
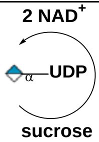	**2**: SuSy, UGDH **3**: 2 + enzyme for NAD^+ regeneration	Sucrose, (NAD^+)	(Engels et al. 2015; Eisele et al. 2018)
**UDP-GlcNAc**			
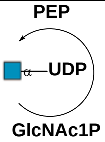	**3**: GlmU, PPase, PK	GlcNAc1P, PEP	(De Luca et al. 1995)
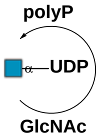	**4:** NaHK; GlmU or AGX1, PPase, PPK	GlcNAc, polyP	(Gottschalk et al. 2021)
**UDP-GalNAc**			
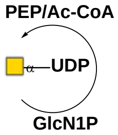	**5**: GlnM, GlmU, PPase, GalE, PK **6**: 5 + enzyme for Ac-CoA regeneration	GlcN1P, PEP, Ac-CoA, (acetyl-P)	(Shao et al. 2002)
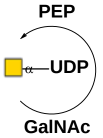	**4**: NaHK; GlmU or AGX1, PPase, PK	GalNAc, PEP	(Tsai et al. 2013)
**GDP-Man**			
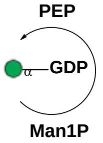	**3**: GDP-Man PP (ManC), PPase, PK	Man1P, PEP	(Wang et al. 1993)
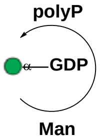	**5**: GlcK, ManB, GDP-Man PP (ManC), PPase, PPK	Man, polyP	(Rexer et al. 2018)

Table S1 continued

Regeneration cycle	Number of enzymes for regeneration of	Stoichiometric substrates or in excess	References
		**GDP-Fuc**	
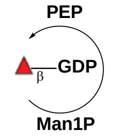	**5**: ManC, PPase, GMD, GDP-Fuc Synthetase, PK<br><br>**6**: 5 + enzyme for NADH regeneration	Man1P, PEP	(Ichikawa et al. 1992b)
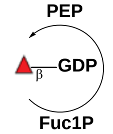	**3**: FKP, PPase, PK	Fuc1P, PEP	(Ichikawa et al. 1992b)
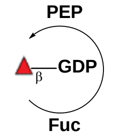	**3**: FKP, PPase, PK	Fuc, PEP	(Tsai et al. 2013)
		**CMP-Neu5Ac**	
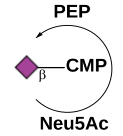	**4**: CSS, PPase, CMPK, PK	Neu5Ac, PEP	(Ichikawa et al. 1991)
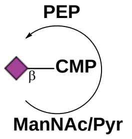	**5**: Neu5Ac aldolase, CSS, PPase, CMPK, PK	ManNAc, PEP	(Liu et al. 1992; Ichikawa et al. 1992a)
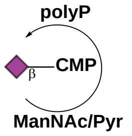	**5**: Neu5Ac aldolase, CSS, PPase, CMPK, PPK	ManNAc, Pyr, polyP	(Nahálka, Pätoprstý 2009)
		**dTDP-/UDP-L-Rha**	
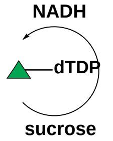	**5 (6):** *St*SuSy, RmlB, RmlC, RmlD, (NOX for NAD^+ regeneration from NADH	sucrose	(Rupprath et al. 2007)
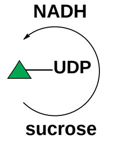	**2:** *Gm*SuSy, *At*RHM1	sucrose	(Thapa et al. 2019)
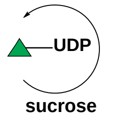	**3**: *Gm*SuSy, *Vv*RHM/AtNRS/ER	sucrose	(Pei et al. 2018)
		**UDP-L-Ara**	
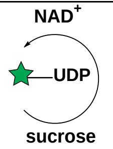	**4**:*Gu*SuS1-Δ9, *At*UGDH3, *At*UX3, *Ps*UGE2	Sucrose, NAD^+	(Sun et al. 2023)

References

Anwar MT, Kawade SK, Huo Y-R, Adak AK, Sridharan D, Kuo Y-T, Fan C-Y, Wu H-R, Lee Y-S, Angata T, Lin C-C (2022) Sugar nucleotide regeneration system for the synthesis of Bi- and triantennary N-glycans and exploring their activities against siglecs. Eur J Med Chem 232:114146. doi:10.1016/j.ejmech.2022.114146

De Luca C, Lansing M, Martini I, Crescenzi F, Shen G-J, O'Regan M, Wong C-H (1995) Enzymic Synthesis of Hyaluronic Acid with Regeneration of Sugar Nucleotides. J Am Chem Soc 117 (21):5869-5870. doi:10.1021/ja00126a034

Eisele A, Zaun H, Kuballa J, Elling L (2018) In Vitro One-Pot Enzymatic Synthesis of Hyaluronic Acid from Sucrose and *N*-Acetylglucosamine: Optimization of the Enzyme Module System and Nucleotide Sugar Regeneration. ChemCatChem 10 (14):2969-2981. doi:10.1002/cctc.201800370

Engels L, Henze M, Hummel W, Elling L (2015) Enzyme Module Systems for the Synthesis of Uridine 5′-Diphospho-α-D-glucuronic Acid and Non-Sulfated Human Natural Killer Cell-1 (HNK-1) Epitope. Adv Synth Catal 357 (8):1751-1762. doi:10.1002/adsc.201500180

Frohnmeyer H, Elling L (2023) Enzyme cascades for the synthesis of nucleotide sugars: Updates to recent production strategies. Carbohydr Res 523:108727. doi:10.1016/j.carres.2022.108727

Gantt RW, Peltier-Pain P, Cournoyer WJ, Thorson JS (2011) Using simple donors to drive the equilibria of glycosyltransferase-catalyzed reactions. Nat Chem Biol 7 (10):685-691. doi:10.1038/nchembio.638

Gottschalk J, Blaschke L, Aßmann M, Kuballa J, Elling L (2021) Integration of a Nucleoside triphosphate regeneration system in the one-pot synthesis of UDP-sugars and hyaluronic acid. ChemCatChem 13 (n/a):3074-3083. doi:10.1002/cctc.202100462

Gygax D, Spies P, Winkler T, Pfaar U (1991) Enzymatic synthesis of β-D-glucuronides with in situ regeneration of uridine 5′-diphosphoglucuronic acid. Tetrahedron 47 (28):5119-5122. doi:10.1016/S0040-4020(01)87124-6

Haynie SL, Whitesides GM (1990) Enzyme-catalyzed organic synthesis of sucrose and trehalose with in situ regeneration of UDP-glucose. Appl Biochem Biotechnol 23 (2):155-170. doi: 10.1007/BF02798384

Hokke CH, Zervosen A, Elling L, Joziasse DH, van den Eijnden DH (1996) One-pot enzymatic synthesis of the Gal(a1-3)Gal(b1-4)GlcNAc sequence with *in situ* UDP-Gal regeneration. Glycoconjugate J 13 (4):687-692. doi:10.1007/BF00731458

Ichikawa Y, Look GC, Wong C-H (1992a) Enzyme-catalyzed oligosaccharide synthesis. Anal Biochem 202 (2):215-238. doi:10.1016/0003-2697(92)90099-S

Ichikawa Y, Liu JL-C, Shen G-J, Wong C-H (1991) A highly efficient multienzyme system for the one-step synthesis of a sialyl trisaccharide: in situ generation of sialic acid and N-Acetyllactosamine coupled with regeneration of UDP-Glc. J Amer Chem Soc 113:6300-6302. doi:10.1021/ja00016a073

Ichikawa Y, Lin YC, Dumas DP, Shen GJ, Garciajunceda E, Williams MA, Bayer R, Ketcham C, Walker LE, Paulson JC, Wong CH (1992b) Chemical-Enzymatic Synthesis and Conformational-Analysis of Sialyl Lewis-X and Derivatives. J Am Chem Soc 114 (24):9283-9298. doi:10.1021/ja00050a007

Jiao R, Zhang L, You R, Peng X, Pei C, Jiang B, Hu M, Li J, Du Y, Qian EW (2024) Efficient and Cost-Effective Synthesis of N-Acetyllactosamine by Sequential Modular Enzymatic Cascade Reactions Involving NTP Regeneration. J Agr Food Chem. doi:10.1021/acs.jafc.4c08638

Liu JLC, Shen GJ, Ichikawa Y, Rutan JF, Zapata G, Vann WF, Wong CH (1992) Overproduction of CMP-Sialic Acid Synthetase for Organic- Synthesis. J Am Chem Soc 114 (10):3901-3910. doi:DOI 10.1021/ja00036a044

Masada S, Kawase Y, Nagatoshi M, Oguchi Y, Terasaka K, Mizukami H (2007) An efficient chemoenzymatic production of small molecule glucosides with in situ UDP-glucose recycling. FEBS Lett 581 (13):2562-2566. doi:10.1016/j.febslet.2007.04.074

Minami A, Kakinuma K, Eguchi T (2005) Aglycon switch approach toward unnatural glycosides from natural glycoside with glycosyltransferase VinC. Tetrahedron Lett 46 (37):6187-6190. doi:10.1016/j.tetlet.2005.07.083

Nahálka J, Pätoprstý V (2009) Enzymatic synthesis of sialylation substrates powered by a novel polyphosphate kinase (PPK3). Org Biomol Chem 7 (9):1778-1780. doi:10.1039/B822549B

Noguchi T, Shiba T (1998) Use of *Escherichia coli* polyphosphate kinase for oligosaccharide synthesis. Biosci Biotechnol Biochem 62 (8):1594-1596. doi:10.1271/bbb.62.1594

Pei J, Chen A, Zhao L, Cao F, Ding G, Xiao W (2017) One-Pot Synthesis of Hyperoside by a Three-Enzyme Cascade Using a UDP-Galactose Regeneration System. J Agr Food Chem 65 (29):6042-6048. doi:10.1021/acs.jafc.7b02320

Pei J, Chen A, Sun Q, Zhao L, Cao F, Tang F (2018) Construction of a novel UDP-rhamnose regeneration system by a two-enzyme reaction system and application in glycosylation of flavonoid. Biochem Eng J 139:33-42. doi:10.1016/j.bej.2018.08.007

Rexer TFT, Schildbach A, Klapproth J, Schierhorn A, Mahour R, Pietzsch M, Rapp E, Reichl U (2018) One pot synthesis of GDP-mannose by a multi-enzyme cascade for enzymatic assembly of lipid-linked oligosaccharides. Biotechnol Bioeng 115 (1):192-205. doi:10.1002/bit.26454

Rupprath C, Kopp M, Hirtz D, Müller R, Elling L (2007) An Enzyme Module System for *in situ* regeneration of dTDP-activated deoxysugars. Adv Synth Catal 349 (8-9):1489-1496. doi:10.1002/adsc.200700058

Shao J, Zhang J, Kowal P, Wang PG (2002) Donor Substrate Regeneration for Efficient Synthesis of Globotetraose and Isoglobotetraose. Appl Environ Microbiol 68 (11):5634-5640. doi:10.1128/AEM.68.11.5634-5640.2002

Sun Q, Guo F, Ren S, Zhang L, Liu X, Li C, Feng X (2023) Construction of a UDP-Arabinose Regeneration System for Efficient Arabinosylation of Pentacyclic Triterpenoids. ACS Synth Biol 12 (8):2463-2474. doi:10.1021/acssynbio.3c00351

Thapa SB, Pandey RP, Bashyal P, Yamaguchi T, Sohng JK (2019) Cascade biocatalysis systems for bioactive naringenin glucosides and quercetin rhamnoside production from sucrose. Appl Microbiol Biotechnol 103 (19):7953-7969. doi:10.1007/s00253-019-10060-5

Tsai T-I, Lee H-Y, Chang S-H, Wang C-H, Tu Y-C, Lin Y-C, Hwang D-R, Wu C-Y, Wong C-H (2013) Effective Sugar Nucleotide Regeneration for the Large-Scale Enzymatic Synthesis of Globo H and SSEA4. J Am Chem Soc 135 (39):14831-14839. doi:10.1021/ja4075584

Varki A, Cummings RD, Aebi M, Packer NH, Seeberger PH, Esko JD, Stanley P, Hart G, Darvill A, Kinoshita T, Prestegard JJ, Schnaar RL, Freeze HH, Marth JD, Bertozzi CR, Etzler ME, Frank M, Vliegenthart JF, Lütteke T, Perez S, Bolton E, Rudd P, Paulson J, Kanehisa M, Toukach P, Aoki-Kinoshita KF, Dell A, Narimatsu H, York W, Taniguchi N, Kornfeld S (2015) Symbol Nomenclature for Graphical Representations of Glycans. Glycobiology 25 (12):1323-1324. doi:10.1093/glycob/cwv091

Wang P, Shen GJ, Wang YF, Ichikawa Y, Wong CH (1993) Enzymes in Oligosaccharide Synthesis - Active-Domain Overproduction, Specificity Study, and Synthetic Use of an alpha-1,2-Mannosyltransferase with Regeneration of GDP-Man. J Org Chem 58 (15):3985-3990. doi:10.1021/jo00067a035

Wong CH, Wang R, Ichikawa Y (1992) Regeneration of Sugar Nucleotide for Enzymatic Oligosaccharide Synthesis - Use of Gal-1-Phosphate Uridyltransferase in the Regeneration of UDP-Galactose, UDP- 2-Deoxygalactose, and UDP-Galactosamine. J Org Chem 57 (16):4343-4344. doi:10.1021/jo00042a008

Wu H-R, Anwar MT, Fan C-Y, Low PY, Angata T, Lin C-C (2019) Expedient assembly of Oligo-LacNAcs by a sugar nucleotide regeneration system: Finding the role of tandem LacNAc and sialic acid position towards siglec binding. Eur J Med Chem 180:627-636. doi:10.1016/j.ejmech.2019.07.046

Zervosen A, Elling L (1996) A novel three-enzyme reaction cycle for the synthesis of *N*- acetyllactosamine with in situ regeneration of uridine 5'- diphosphate glucose and uridine 5'-diphosphate galactose. J Am Chem Soc 118 (8):1836-1840. doi:10.1021/ja953495e
